# Systematic identification of autophagy-related proteins in *Aedes albopictus*

**DOI:** 10.1371/journal.pone.0245694

**Published:** 2021-01-19

**Authors:** Yu Wang, Jialu Qiao, Dandan Zhang, Chunyan Zhong, Shengya Wang, Xiaomei Li, Lingyan Feng, Shen Shi, Bingxue Wang, Qingzhen Liu

**Affiliations:** State Key Laboratory of Virology, Modern Virology Research Center, College of Life Sciences, Wuhan University, Wuhan, Hubei, China; Chinese Academy of Sciences, CHINA

## Abstract

Autophagy is a conserved cellular process playing a role in maintenance of cellular homeostasis and response to changing nutrient conditions via degradation and recirculation of cellular redundant components. Autophagy-related proteins (Atg) play important function in autophagy pathway. *Aedes albopictus* mosquito is an effective vector transmitting multiple viruses which cause serious human diseases. Moreover, *Aedes albopictus* mosquito is becoming a serious threat to human health due to its widening distribution in recent years and thus worth of more research attention. It was reported that autophagy might play a role in viral infection in *Aedes* mosquito. To better understand the interaction between autophagy and arbovirus infection in mosquito system, it is necessary to identify autophagy pathway in the system. However, autophagy in *Aedes albopictus* mosquito is still poorly understood so far. We recently identified AaAtg8, the first Atg protein reported in *Aedes albopictus* mosquito. This work further identified twelve *atg* genes in *Aedes albopictus* mosquito. Sequence and phylogenetic analysis of the twelve *atg* genes were performed. Expression profiles of all the twelve *Aaatg* genes in different developmental stages and genders of *Aedes albopictus* mosquito were conducted. Effects of chemicals inhibiting or inducing autophagy on the levels of eight identified AaAtg proteins were examined. The function of two identified AaAtg proteins AaAtg6 and AaAtg16 and their response to arbovirus SINV infection were studied preliminarily. Taken together, this work systematically identified *Aedes albopictus atg* genes and provided basic information which might help to elucidate the autophagy pathway and the role of autophagy in arbovirus infection in *Aedes* mosquito system.

## Introduction

Autophagy is a highly conserved cellular process removing redundant proteins and damaged organelles which plays an important role in maintaining cell homeostasis and responding to changing nutrient conditions [1]. Autophagy also acts as a cellular defense mechanism against pathogenic bacteria, parasites and virus [2,3]. According to the way to deliver cargo, autophagy can be divided into macroautophagy, microautophagy and chaperone mediated autophagy [4]. Microautophagy refers to the direct phagocytosis of specific organelles through the endocytosis of lysosome membrane or vacuole surface [5]. Chaperone mediated autophagy recognizes specific protein substrates through cytoplasm chaperones and delivers the substrates as unfolded soluble proteins directly to lysosomes for degradation [6]. Macroautophagy involves membrane rearrangement. It is the most commom form of autophagy. Hereinafter, autophagy is referred to macroautophagy.

During macroautophagy, a small part of the cytoplasm is sequestered by a membrane sac named isolation membrane or phagophore, phagophore elongates and subsequently encloses to form a double-membrane structure termed autophgosome. The outer membrane of the autophagosome then fuses with lysosome to form autolysosome. After fusion, the inner membrane and enclosed cytoplasmic materials are degraded by lysosomal enzymes and the degradation products are delivered back to cytoplasm for recycling [7]. Autophagy-related proteins (Atg) play critical functions during autophagy process. So far, more than 40 autophagy genes have been identified in yeast [8]. Homologues of yeast Atg proteins are also found in other species including insects and mammals [9]. Atg proteins involved in autophagy can be divided into the following groups: (1) ULK1/Atg1 complex mainly composed of Atg1, Atg13, Atg17 and Atg101 (or Atg29 and Atg31 in yeast), regulates the initiation stage of autophagosome formation; (2) Atg9 complex containing Atg2, Atg9 and Atg18 functions as a carrier in supplying membrane; (3) PI3K complex containing Vps34, Vps15, Atg6 and Atg14 plays a role in the process of vesicle nucleation and regulates autophagy by producing PI3P; (4) Two kinds of ubiquitin-like conjugation systems, namely the Atg12-Atg5-Atg16 complexes and the Atg8 system take part in extension of the autophagosome membrane and formation of complete vesicles [9].

*Aedes albopictus* mosquito is an effective vector for transmission of multiple virus including dengue viruses (DENV), zika viruses (ZIKA) and chikungunya viruses (CHIKV) which cause serious human disease [10–12]. Moreover, *Aedes albopictus* mosquito, native in Southeast Asia, is spreading rapidly around the world in recent years. Thus, it is becoming an increasingly important threat to public health [13]. Despite a series of studies, there is still no effective vaccine or treatment for the diseases caused by viruses transmitted via *Aedes albopictus* mosquito [14]. Recently, it is reported that apoptosis related genes control autophagy and influence type 2 dengue virus infection in *Aedes aegypti* mosquito, suggesting a possible role of autophagy in virus infection in *Aedes* mosquito [15]. Therefore, elucidation of the role of autophagy in viral infection in *Aedes* mosquito might provide a new angle to inhibit vector competence of *Aedes* mosquito. However, autophagy in *Aedes albopictus* is still poorly understood.

The first *atg* gene *Aaatg8* in *Aedes albopictus* mosquito was identified in 2018 by our group. In this study, twelve novel *atg* genes were identified in *Aedes albopictus*. Sequence alignment and phylogenetic analysis of the identified genes revealed that all the twelve genes belonged to *atg* gene family. Expression profiles of the twelve genes during development and adult stages were conducted. Also, effects of autophagy inhibitors and inducers on the levels of eight identified AaAtg proteins were examined. The function of two identified AaAtg proteins and their response to arbovirus Sindbis virus (SINV) infection were studied preliminarily. The information provided in this study may help the understanding of autophagy mechanism in *Aedes albopictus* mosquito.

## Materials and methods

### Cells and mosquitoes

C6/36 cells generated from larvae of *Aedes albopictus* were provided by China Center for Type Culture Collection (CCTCC) and were maintained in minimal essential medium (MEM HyClone) supplemented with 10% fetal bovine serum (FBS, Biological Industries) at 28°C with 5% CO_2_.

*Aedes albopictus* were maintained in our laboratory. Larvae and pupae were fed on mixture of finely ground cat food. *Aedes albopictus* mosquitoes were fed on 10% sucrose and reared at 28°C with approximately 80% relative humidity under a 16: 8 light: dark photoperiod.

### Chemical reagents and antibodies

3-MA (5mM, dissolved in ddH2O) and CQ (300μM, dissolved in ddH2O) were purchased from Sigma-Aldrich, and Rapa (500nM, dissolved in DMSO) was purchased from MedChemExpress. The primary and secondary antibodies used in the study include purchased commercial mouse antibody against actin (Proteintech), rabbit antibody against Atg8 (GABARAP, Medical & Biological Laboratories), rabbit antibody against Atg6 (Beclin1, Medical & Biological Laboratories), anti-mouse IgG-HRP secondary antibody (Thermo Fisher Scientific) and anti-rabbit IgG-HRP secondary antibody (Thermo Fisher Scientific) and polyclonal rabbit antibody against AaAtg1, AaAtg3, AaAtg5, AaAtg9, AaAtg10, AaAtg16 and AaAtg101 prepared by our laboratory.

### RNA preparation and cDNA synthesis

Total RNA from C6/36 cells and *Aedes albopictus* mosquitoes were isolated using TRIzol reagent (Invitrogen) according to the protocol provided by the manufacturer. Briefly, after homogenizing the sample with TRIzol, chloroform was added and homogenate was separate into three layers after centrifugation. The RNA in the upper aqueous layers was precipitated with isopropanol, followed by washing with 75% ethanol to remove impurities and dissolved in RNase-free water. After digestion of genomic DNA by DNase I (Fermentas) from prepared RNA sample, 1 ug of total RNA was used in the first strand cDNA synthesis by using M-MLV Reverse Transcriptase (Invitrogen) as described in protocol by manufacturer. For mosquitoes, the samples were washed three times by RNase-free water, After homogenizing in TRIzol reagent by grinding, the samples were centrifuged at 12000 g for 10 min at 4°C to remove insoluble material and subjected to RNA and cDNA preparation as above.

### Identification of *Aaatg* cDNA

Using primers designed according to *Aedes aegypti atg* genes, fragments of the *atg* genes of *Aedes albopictus* were amplified from C6/36 cDNA by PCR. The PCR products were cloned into pCR-II vector (TA Cloning^®^ Kit; Invitrogen, CA, USA) and sequenced by Sangon Biotech (Shanghai). The obtained sequences were used to design primers for rapid amplification of cDNA ends (RACE). 5’ and 3’ RACE were operated with SMARTer^™^ RACE cDNA Amplification Kit (Clontech, CA, USA). The 5’ and 3’RACE products were cloned into pCR-II vector and subjected to sequencing. The ORF of each *atg* gene was amplified from C6/36 cDNA by PCR using primers designed by sequences of RACE products and cloned into pCR-II followed by sequencing. The sequencing results from positive colonies confirmed the sequence information obtained from sequencing of RACE products. The primer sequences used here are listed in S1 Table.

### Synthesis of siRNA and transfection

The siRNA was designed using siDirect 2.0 Web server (http://sidirect2.rnai.jp/). The 21 nt sense and antisense RNA oligo sequences were synthesized by Sangon Biotech (Shanghai). The sequences used here are list in S2 Table.

C6/36 cells were transfected using lipofectamin 2000 reagent (Invitrogen) according to the manufacturer’s instructions when the cells grown to 80% confluence in the culture plate. A total of 100 pmol siRNA dissolved in 60 μL of opti-MEM was incubated with 2μL lipofectain dissolved in 60 μL of opti-MEM at room temperature for 30 min before added to the cells in 12-wells plates. The medium was changed after 6 hours post transfection, and the cells were cultured in fresh medium up to the indicated time.

### SINV infection

SINV infection of C6/36 cells (MOI of 5) was performed in 28°C with 5% CO_2_. SINV-TE virus was diluted in serum-free medium and added to C6/36 cells. After 2 hours absorption, the medium was removed and the cells were washed with PBS and then cultured in fresh medium up to the indicated time.

### Quantitative real-time PCR

The qRT-PCR experiment was conducted by using a CFX96 real-time PCR detection system (Bio-Rad) with SYBR green dye (Invitrogen). Relative level of specific gene transcription was determined by normalization to ribosomal gene *s7* (*Aas7*; Genebank: JN132168.1). Quantity values were generated by using 2^-ΔΔCt^ method as described previously [16]. The primer sequences used here are listed in S1 Table.

### Western blotting analysis

Prepared cell lysates were separated by SDS-PAGE after boiled at 100°C for 5 min with 5 × SDS Loading buffer and transferred to Immobilon-P membrane (Merck Millipore). The membrane was blocked with 5% skimmed milk in Tris-buffer saline with 0.1% Tween 20. Then the membrane was incubated with indicated primary antibody, followed by a secondary antibody conjugated with horseradish peroxidase (HRP). Imaging was detected in LAS 4000 (Fujifilm) after incubating in an enhanced chemiluminescence reagent (Merck Millipore) for 1 min. The images’ brightness and contrast were adjusted using Multi Gauge software and the backgrounds were subtracted using ImageJ. To measure the integrated densities, the intensities of regions of interest were selected for all bands and normalized to that of corresponding Actin.

## Results

### Sequence analysis of AaAtg proteins

**Fig 1:**
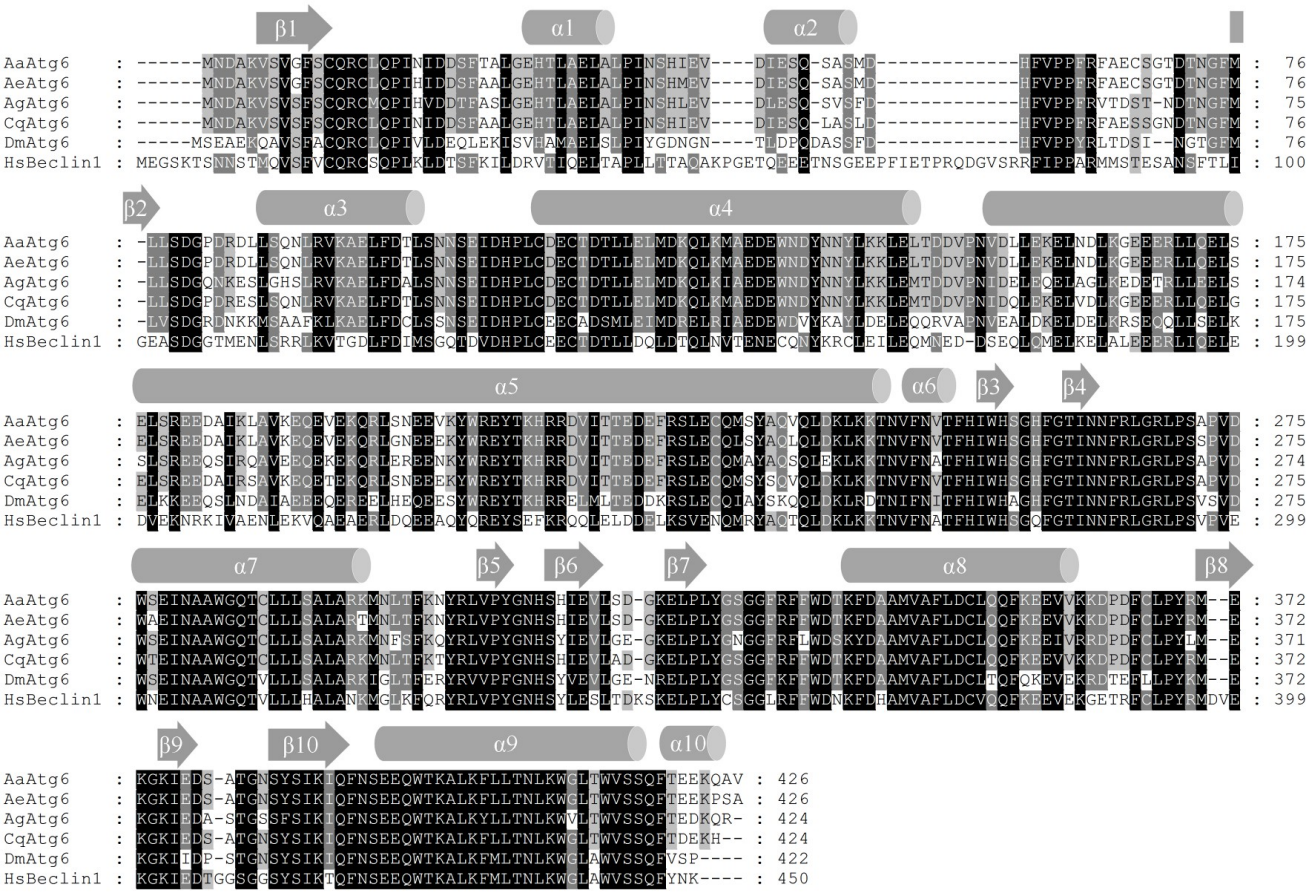
Sequence analysis of AaAtg6. The amino acid sequence of AaAtg6 was shown in alignment with Atg6 orthologs from *Aedes aegypti* (AeAtg6, NCBI: XP_001654535.1), *Anopheles gambiae* (AgAtg6, NCBI: XP_310418.5), *Culex quinquefasciatus* (CqAtg6, XP_001861442.1), *Drosophila melanogaster* (DmAtg6, NCBI: NP_651209.1) and *Homo sapiens* (HsBeclin1, NCBI: NP_001300927.1). The alignment was performed by using ClustalX 2.1 and modified by GeneDoc 3.2. The amino acid residues identical among 6, 5 and 4 or 3 orthologs were indicated by white letters within black boxes, white letters within dark gray boxes, and black letters within light gray boxes, respectively. Secondary structures were predicted using PSIPRED 4.0. α: alpha helices; β: beta sheets.

Sequence information of twelve *Aedes albopictus atg* genes, namely, *Aaatg1* (MT921132), *Aaatg3* (MT921133), *Aaatg4* (MT921134), *Aaatg5* (MT921135), *Aaatg6* (MT921136), *Aaatg7* (MT921137), *Aaatg9* (MT921138), *Aaatg10* (MT921139), *Aaatg12* (MT921140), *Aaatg13* (MT921141), *Aaatg16* (MT921142), *Aaatg101* (MT921143), was obtained according to the method described in materials and methods. The sequences of all the identified AaAtg proteins were analyzed. Take *Aaatg6* as an example, the ORF of *Aaatg6* encoded a putative protein containing 426 amino acids. AaAtg6 shared 97%, 94%, 83%, 62% and 51% sequence identity with Atg6 orthologs of *Aedes aegypt*, *Culex quinquefasciatus*, *Anopheles gambiae*, *Drosophila melanogaster* and *Homo sapiens*, respectively. The predicted secondary structure of AaAtg6 consisted of a series of alpha helices and beta sheets that were highly conserved in other Atg6 orthologs (Fig 1). Multiple-sequence alignment and identity analysis of other eleven identified AaAtg proteins were also conducted with *Aedes Aegypti*, *Anopheles gambiae*, *Culex quinquefasciatus*, *Drosophila melanogaster* and *Homo sapiens* (S1–S11 Figs). The results revealed that every identified AaAtg shared the highest amino acid sequence identity with its ortholog of *Aedes aegypti*.

### Phylogenetic analysis of AaAtg proteins

**Fig 2:**
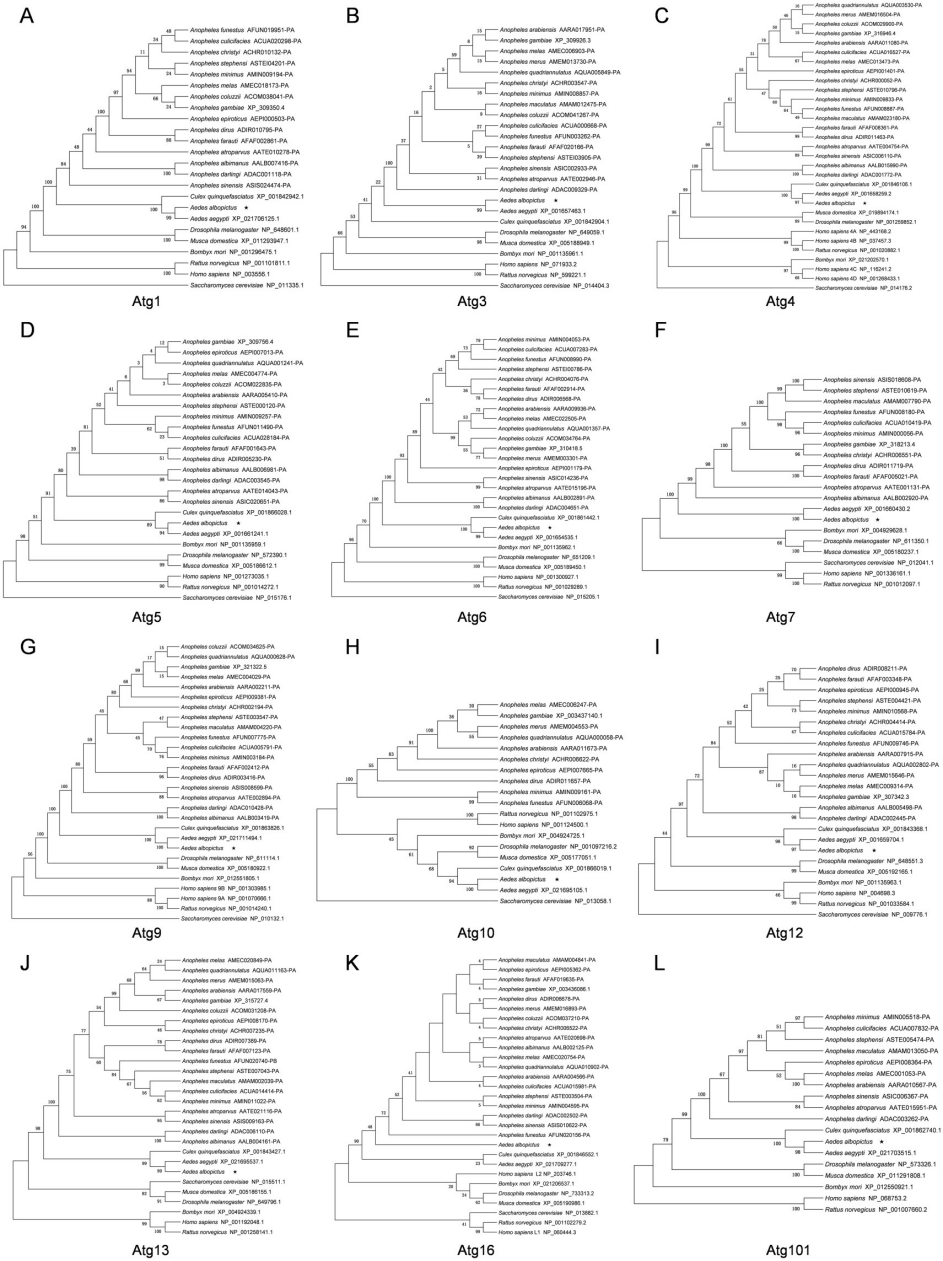
Phylogenetic analysis of AaAtg proteins. (A)—(L) The phylogenetic trees were constructed by MEGA 5.2 using the neighbor-joining method and 1000 bootstrap replicates. Multiple sequence alignments were performed using sequences of Atg orthologous from *Saccharomyces cerevisiae*, *Homo sapiens*, *Rattus norvegicus*, *Drosophila melanogaster*, *Musca domestica*, *Bombyx mori* and mosquitoes (*Aedes*, *Anopheles*, *Culex*). The sequences of AaAtg proteins of *Aedes albopictus* were indicated by a pentacle.

The phylogenetic tree of twelve AaAtg proteins were constructed by neighbor-joining method using alignment result of Atg orthologous sequences of mosquitoes (*Aedes*, *Anopheles*, *Culex*) from VectorBase database and *Saccharomyces cerevisiae*, *Homo sapiens*, *Rattus norvegicus*, *Drosophila melanogaster*, *Musca domestica*, *Bombyx mori* from NCBI database (Fig 2). Based on the phylogenetic analysis of the twelve AaAtg proteins, *Aedes albopictus* formed a single clade inside the mosquitoes group with *Aedes aegypti*. Interestingly, AaAtg13 is different when analysis the evolutionary relationship with mammals. All of AaAtg proteins except AaAtg13 had a much closer relativity to the Atg orthologous of mammals than that of *Saccharomyces cerevisiae*.

### Expression profiles of *Aaatg* genes in developmental and adult stage of *Aedes albopictus*

**Fig 3:**
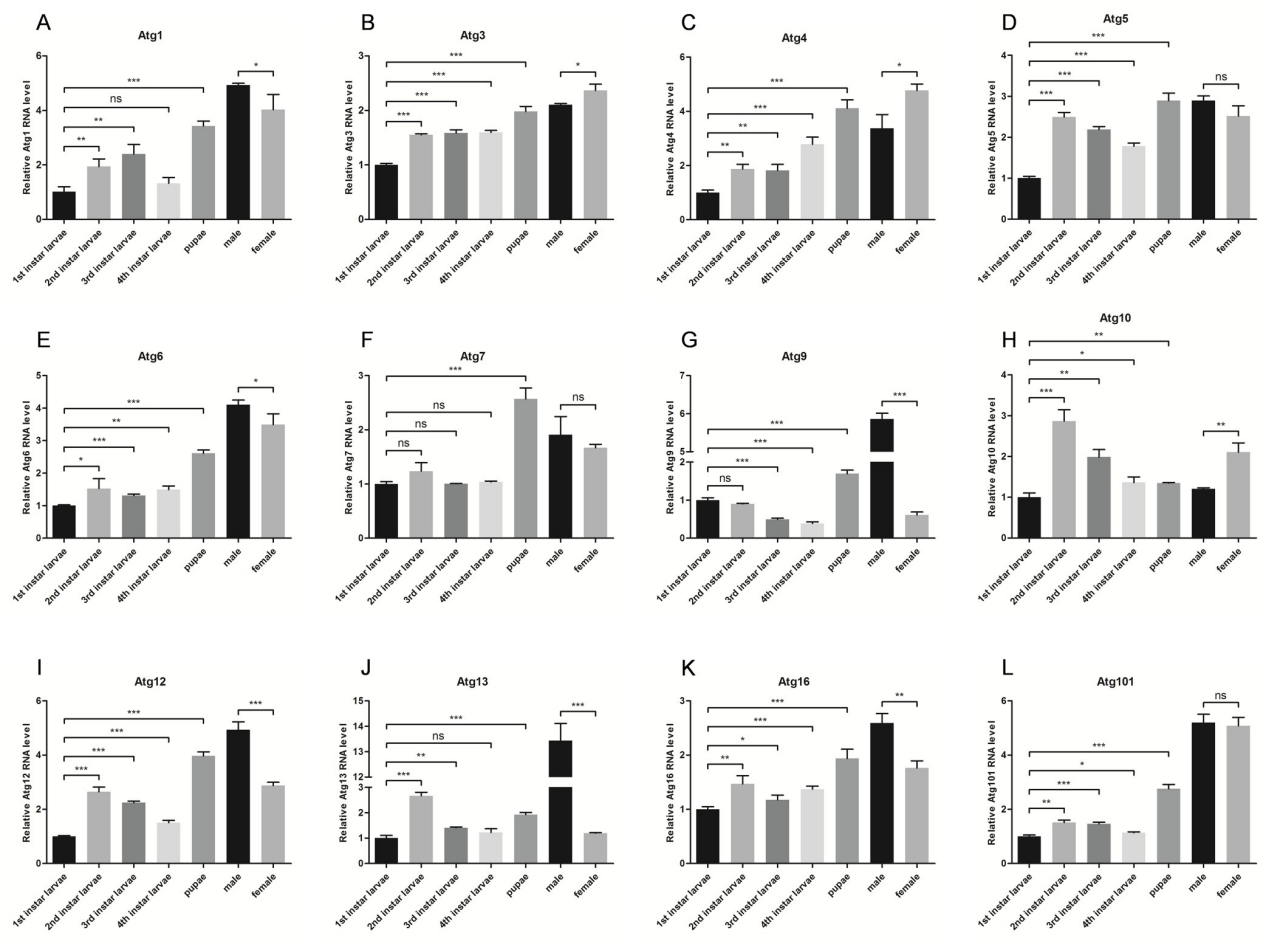
Expression profiles of *Aaatg* genes in developmental and adult stages of *Aedes albopictus*. (A)—(L) RNAs were prepared from 1st to 4th instar larvae, pupae, male and female adults and subjected to qPCR analysis. The vertical axis represents the relative expression level of *Aaatg* gene in different development stages or genders to housekeeping gene *Aas7*. Data were presented as means ± SD for three biological replicates. The statistical significances were calculated by t test, ns P > 0.05, * P < 0.05, **P < 0.01, ***P < 0.001.

In order to explore the expression profiles of *Aaatg* genes in *Aedes albopictu*s mosquito, qRT-PCR were performed to quantify the transcriptional level of all identified *atg* genes in 1st to 4th instar larvae, pupae, male and female adults. *Aedes albopictus* ribosomal gene *s7* (*Aas7*, GeneBank: JN132168.1) was used as a control. All of the twelve *atg* genes were ubiquitously expressed in all development stages (Fig 3). The transcription levels of *Aaatg5*, *Aaatg7*, *Aaatg9*, *Aaatg10*, *Aaatg12*, *Aaatg1*3 and *Aaatg101* were rapidly increased at some point from 1st instar larvae to pupae, while those of *Aaatg1*, *Aaatg3*, *Aaatg4*, *Aaatg6* and *Aaatg1*6 were smoothly changed in larvae and pupae. Besides, the transcription levels of *Aaatg10*, *Aaatg12* and *Aaatg16* were different in male and female, the levels of *Aaatg*9 and *Aaatg13* in female were significantly lower than their counterparts in male.

### Responses of *Aedes albopictus* Atg proteins to chemicals inducing or inhibiting autophagy

**Fig 4:**
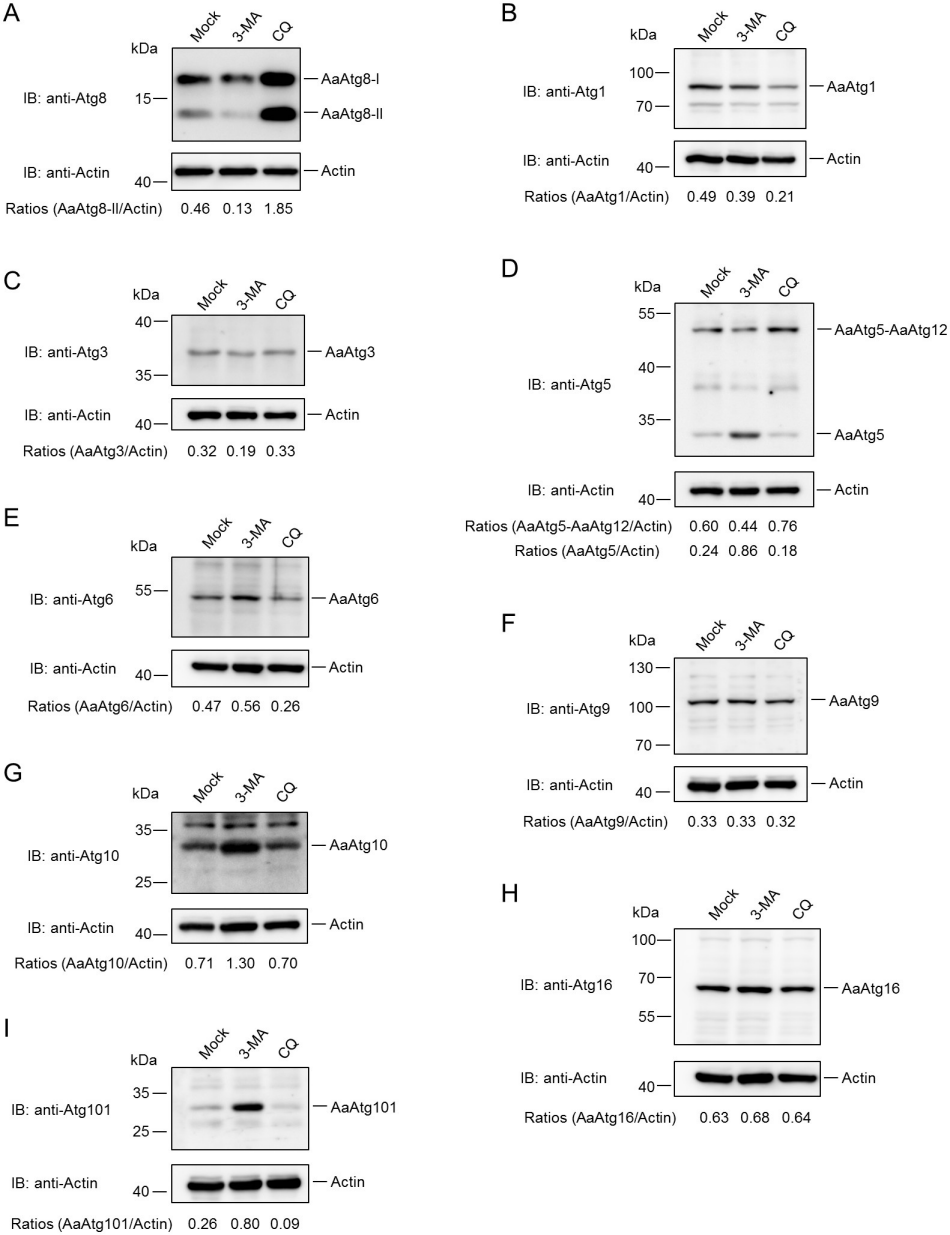
Responses of AaAtg proteins to 3-MA and CQ. (A)—(I) C6/36 cells were mocked treated or treated with 3-MA (5mM, dissolved in ddH_2_O) or CQ (300μM, dissolved in ddH_2_O) for 36 hours, respectively. The protein levels of AaAtg8, AaAtg1, AaAtg3, AaAtg5, AaAtg6, AaAtg9, AaAtg10, AaAtg16, AaAtg101 and actin were analysed by Western Blotting, respectively. The relative level of each AaAtg protein to that of actin was quantified by ImageJ according to materials and methods.

To investigate the properties of identified autophagy proteins, responses of *Aedes albopictus* Atg proteins to chemicals inducing or inhibiting autophagy were examined. First responses of AaAtg proteins to 3-methyladenine (3-MA) and Chloroquine (CQ) were tested. 3-MA can inhibit autophagy at early stage by suppression of class III PI3K [17]. CQ inhibits autophagy at late stage by blocking the fusion of autophagosomes and lysosomes [18]. Our previous research has shown that AaAtg8 could act as a marker of autophagy in *Aedes albopictus* C6/36 cells [19]. Thus, the change in the amount of AaAtg8 in C6/36 cells caused by 3-MA or CQ treatment was analysed. It was observed that 3-MA slightly reduced AaAtg8-I and significantly reduced AaAtg8-II, while CQ increased both AaAtg8-I and AaAtg8-II obviously (Fig 4A). The responses of other identified AaAtgs to 3-MA and CQ treatment were also monitored. The results showed that both 3-MA and CQ treatment led to reduction of AaAtg1, and the reduction was more significant in CQ treatment (Fig 4B). The amount of AaAtg3 was decreased by 3-MA, but not affected by CQ treatment (Fig 4C). 3-MA treatment decreased the amount of AaAtg5-AaAtg12 complex, but increased the amount of AaAtg5 monomer, suggesting that 3-MA blocked the formation of AaAtg5-AaAtg12 complex. In contrast, CQ treatment resulted in the increase of AaAtg5-AaAtg12 complex and the reduction of AaAtg5 monomer (Fig 4D). The amount of AaAtg6 was increased by 3-MA and decreased by CQ treatment (Fig 4E). The amount of AaAtg9 was not affected by 3-MA or CQ (Fig 4F). AaAtg10 was significantly increased by 3-MA, but not affected with CQ (Fig 4G). AaAtg16 did not change obviously under the treatment of 3-MA or CQ (Fig 4H). AaAtg101 was significantly increased by 3-MA but decreased with CQ (Fig 4I).

**Fig 5:**
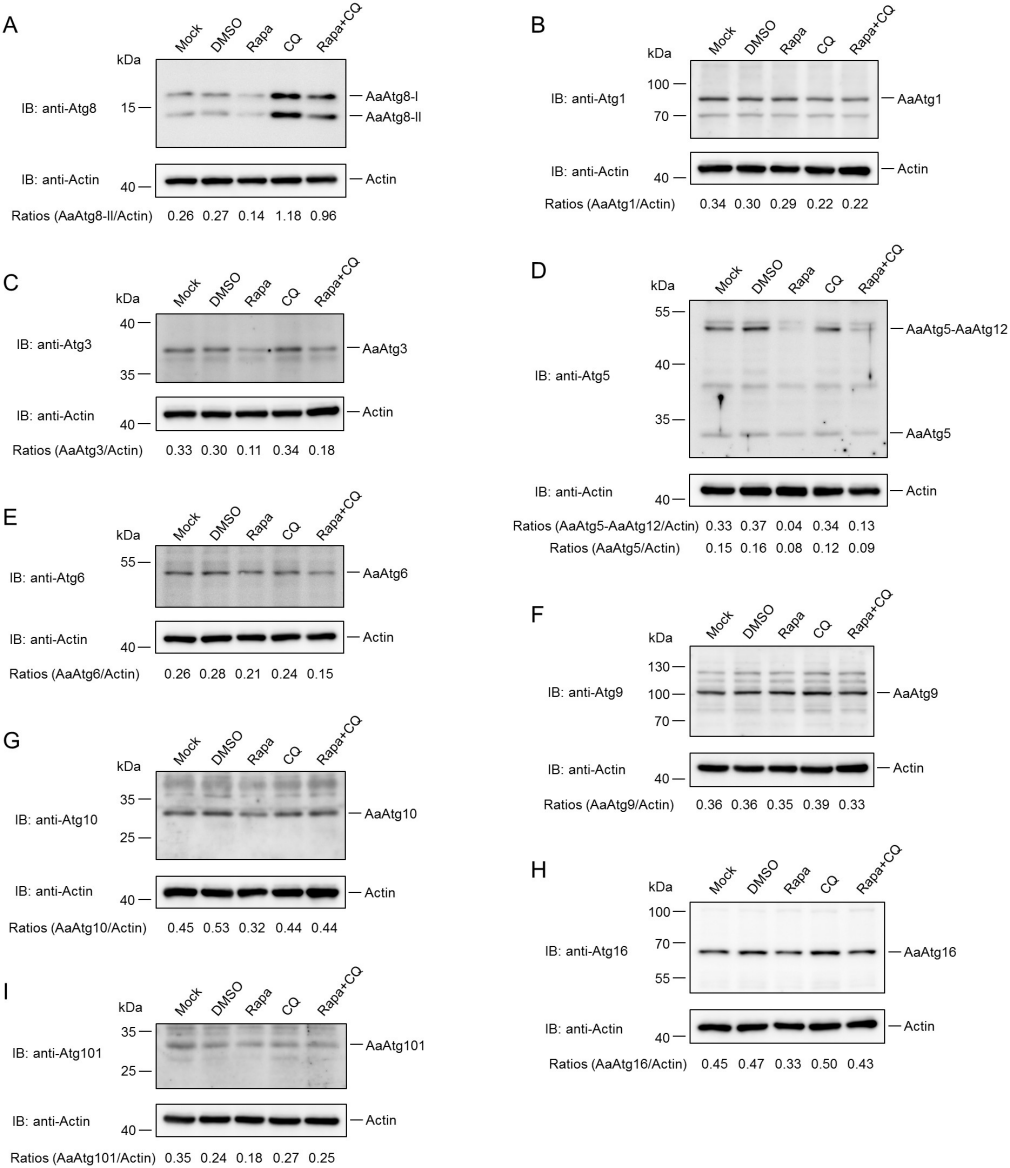
Responses of AaAtg proteins to autophagy Rapa. (A)—(I) C6/36 cells were treated with Rapa (500nM, dissolved in DMSO) or CQ (300μM, dissolved in ddH_2_O) for 24 hours and 12 hours, respectively. For Rapa plus CQ treatment, C6/36 cells were treated with Rapa for 12 hours then treated with CQ for another 12 hours. Mock treated or DMSO treated C6/36 cells were used as controls. The protein levels of AaAtg8, AaAtg1, AaAtg3, AaAtg5, AaAtg6, AaAtg9, AaAtg10, AaAtg16, AaAtg101 and actin were analysed by Western Blotting, respectively. The relative level of each AaAtg protein to that of actin was quantified by ImageJ according to materials and methods.

Rapamycin (Rapa) is a kind of autophagy inducer which induces autophagy by inhibiting TOR complex 1 [20]. How AaAtgs react to Rapa treatment was examined as well. It was observed that both AaAtg8-I and AaAtg8-II decreased by Rapa treatment alone. However a follow up treatment of CQ after Rapa treatment increased both AaAtg8-I and AaAtg8-II (Fig 5A). We hypothesized that Rapa accelerated intracellular autophagic flux, resulting in faster transformation of AaAtg8-I and rapid degradation of AaAtg8-II. The follow up CQ treatment blocked the integrity of autophagic flux and thus weaked the capacity of lysosomal degradation, resulting in increased amount of AaAtg8-I and AaAtg8-II. The detection of other AaAtgs showed that Rapa did not significantly affect AaAtg1 and AaAtg9 (Fig 5B and 5F). But it decreased AaAtg3, AaAtg5-AaAtg12, AaAtg5, AaAtg6, AaAtg10, AaAtg16 and AaAtg101 (Fig 5C–5I). Among them, only AaAtg6 was further reduced after the addition of CQ, because AaAtg6 itself was obviously inhibited by CQ (Fig 4E).

### Atg6 and Atg16 expression levels were influenced by SINV infection

**Fig 6:**
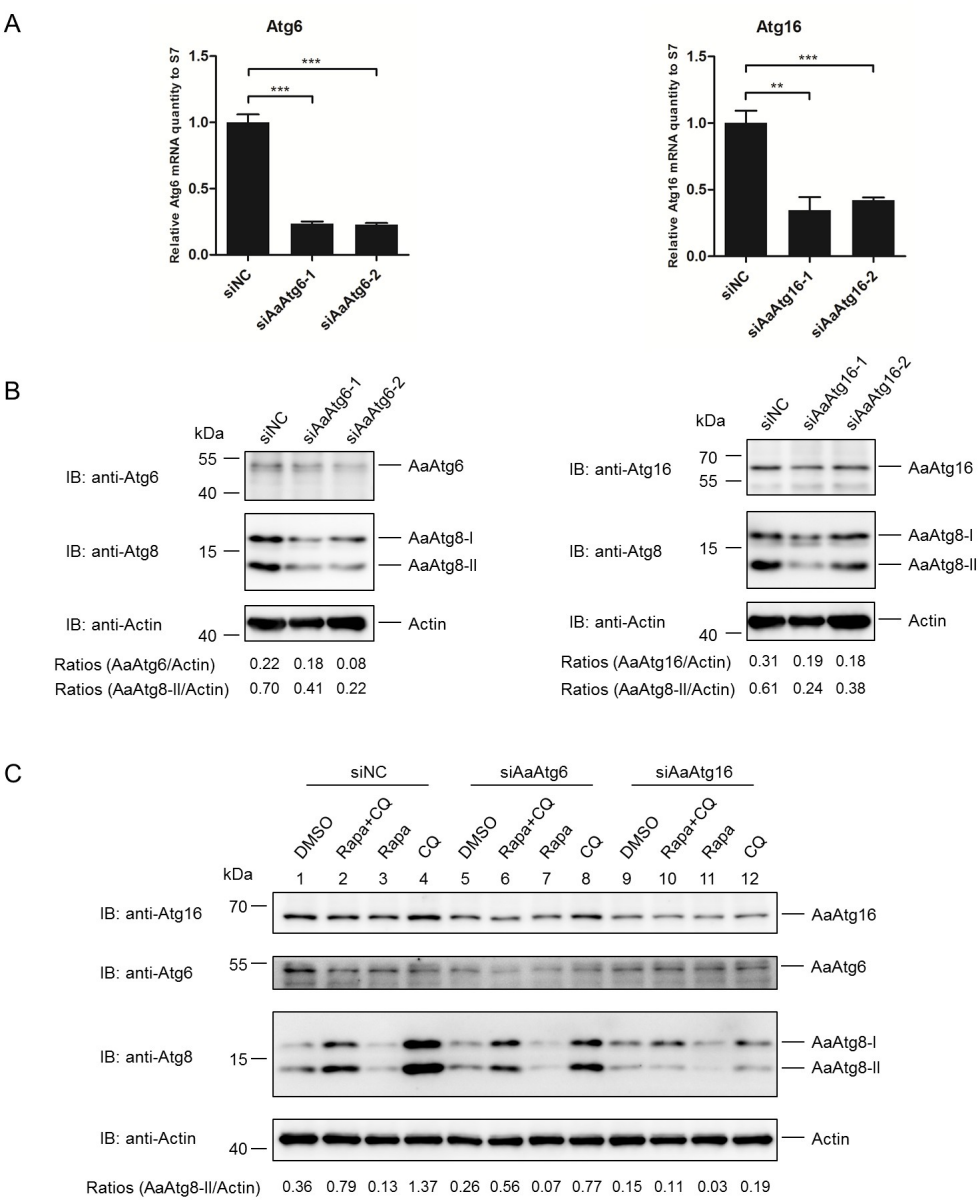
AaAtg6 and AaAtg16 were involved in Rapa induced autophagy. (A) C6/36 cells transfected with equal amounts of RNA of siNC, siAaAtg6-1, siAaAtg6-2, siAaAtg16-1 or siAaAtg16-2 for 48 hours, respectively. RNAi efficiency of AaAtg6 and AaAtg16 were quantified by qPCR analysis. (B) Lysates prepared from C6/36 cells primary treated as described in (A), and subjected to Western Blotting using antibody against AaAtg6, AaAtg16 and AaAtg8, respectively. (C) C6/36 cells transfected with siNC, siAtg6-2 or siAtg16-1 for 24 hours then treated with Rapa or CQ for 24 hours and 12 hours, respectively. For Rapa plus CQ treatment, C6/36 cells were treated with Rapa for 12 hours then treated with CQ for another 12 hours. The protein levels of AaAtg16, AaAtg6, AaAtg8 and actin were analysed by Western Blotting, respectively. The relative level of AaAtg8-II to that of actin was quantified by ImageJ according to materials and methods. Data were presented as means ± SD for three biological replicates. The statistical significances were calculated by t test, **P < 0.01, ***P < 0.001.

It is known that Atg6 and Atg16 are two important Atg proteins functioning in initiation and elongation phase of autophagy, respectively. Atg6 forms part of class III PI3K complex involved in autophagy initiation and phagophore nucleation [21,22], while Atg16 is a component Atg12-Atg5-Atg16 complex mediating conjugation of phosphatidylethanolamine (PE) to LC3 on autophagosomes and thus participates in phagophore expansion [23,24]. To provide useful information for the study of autophagy in C6/36 cells, preliminary investigation of the function of AaAtg6 and AaAtg16 was performed. First, AaAtg6 and AaAtg16 were silenced by siRNA and the effects of the silencing on autophagy in C6/36 cells were examined. The siRNA used in this work efficiently down regulated AaAtg6 and AaAtg16 expression, as verified by quantitative PCR (qPCR) on RNA level and through Western Blotting on protein level (Fig 6A and 6B). In the meantime, knockdown of AaAtg6 and AaAtg16 both let to reduction level of AaAtg8 (Fig 6B), suggesting that AaAtg6 and AaAtg16 are functional Atg proteins in C6/36 cells. To confirm the result, C6/36 cells were subjected to Rapa treatment to induce autophagy after AaAtg6 or AaAtg16 was knockdown by siRNA. As shown, lower levels of AaAtg8 were observed in AaAtg6 and AaAtg16 knockdown C6/36 cells compared to controls, indicating that AaAtg6 and AaAtg16 knockdown reduced the autophagic flux both of basal level autophagy (Fig 6C, lane 1, 5 and 9) and Rapa triggered autophagy (Fig 6C, lane 3, 7 and 11) in C6/36 cells. It is known that CQ blocks the late stage of autophagy and thus causes accumulation of AaAtg8-II generated earlier in autophagy. It was observed that when C6/36 cells were treated with CQ after Rapa treatment, lower levels of AaAtg8 were observed in AaAtg6 and AaAtg16 knockdown C6/36 cells compared to controls (Fig 6C, lane 2, 6 and 10). This result proved that the lower level of AaAtg8-II by Rapa treatment observed in AaAtg6 and AaAtg16 knockdown C6/36 cells were the result of lower level of autophagic flux but not fast degradation of AaAtg8-II by too strong autophagic flux. Taken together, the above data proved that AaAtg6 and AaAtg16 were involved in autophagy induced by Rapa.

**Fig 7:**
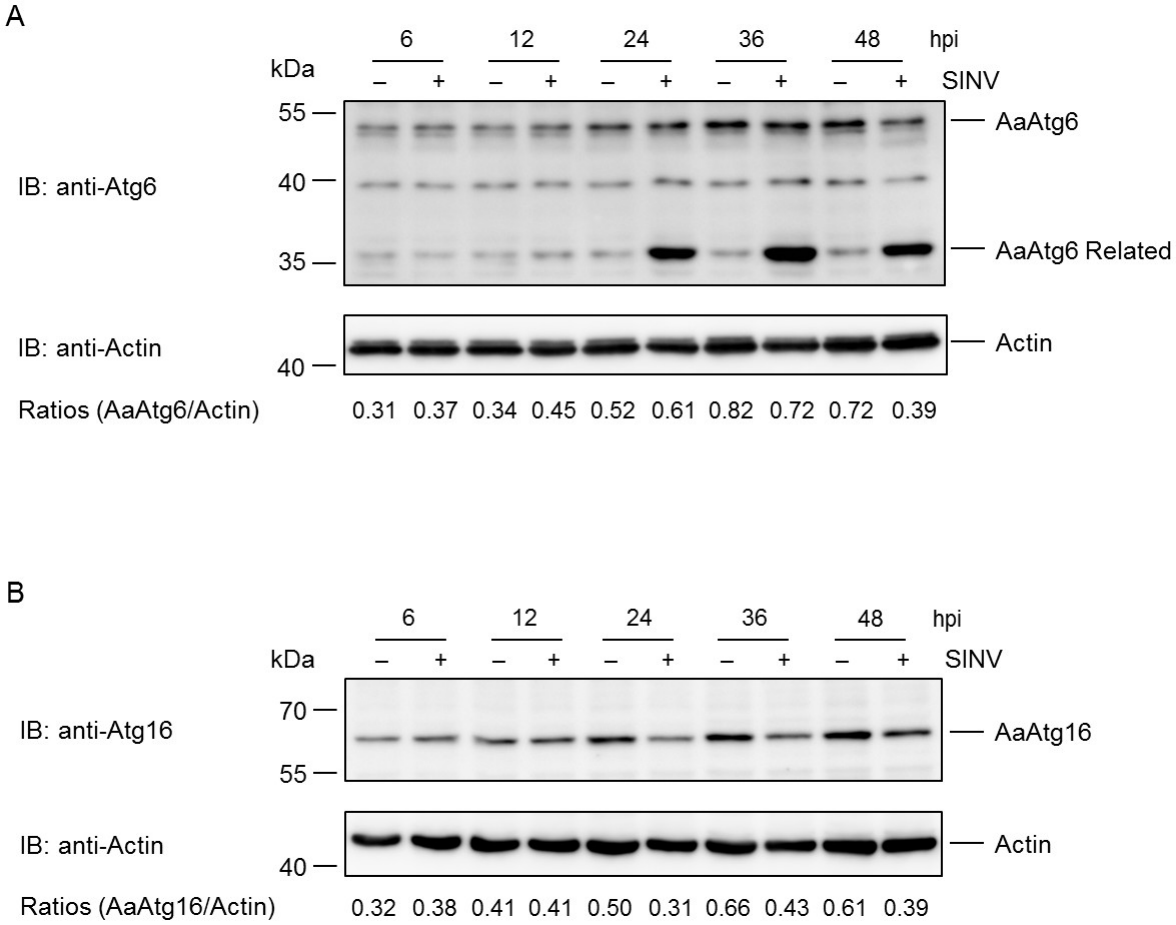
Protein level of AaAtg6 and AaAtg16 at different time points post SINV infection. C6/36 cells were infected with SINV at MOI of 5. (A) and (B) The protein level of AaAtg6 and AaAtg16 at different time points post infection were analysed by Western Blotting, respectively. The relative level of AaAtg6 and AaAtg16 to that of actin was quantified by ImageJ according to materials and methods.

It is known that Atg proteins regulated the translation of viral and antiviral proteins in SINV infection mammalian cells [25,26]. Since it is proved that AaAtg6 and AaAtg16 were functional Atg proteins in C6/36 cells, it is worth to know how they respond to arbovirus infection. To that purpose, C6/36 cells were infected by SINV, a kind of arbovirus transmitted by *Aedes albopictus* and the levels of AaAtg6 and AaAtg16 were monitored. The result showed that the level of AaAtg6 were increased by SINV infection during 6–24 hours post infection but began to decrease after 36 hours post infection (Fig 7A). Remarkably, an AaAtg6 related protein band with lower molecular mass was observed from 24 hours post infection (Fig 7A). Different from AaAtg6, level of AaAtg16 were not significantly influenced by SINV infection during 6–12 hours post infection but began to decrease after 24 hours post infection (Fig 7B). The implication of the above data requires further investigation.

## Discussion

*Aedes albopictus* mosquito transmitting multiple arboviruses is a serious threat to human health. It is known that in mammals, autophagy plays a role either in host antiviral defend or contribution to arbovirus replication [27–29]. But mammals does not replicate the unique physiological feature associated with hematophagous insects like mosquitoes [30]. Moreover, it is recently reported that autophagy might play a role in arbovirus infection in *Aedes* mosquito [31,32]. Therefore, it is significant to elucidate autophagy pathway in *Aedes* mosquito. We have published the first autophagy related gene *Aaatg8* of *Aedes albopictus* mosquito in 2018. This study further identified another twelve autophagy related genes of *Aedes albopictus* mosquito and provided the basic characters of the identified genes.

It has been reported that autophagy genes are required for normal dauer morphogenesis and life-span extension in *C*. *elegans* [33]. In *Drosophila melanogaster*, autophagy is required for developmental salivary gland degradation and midgut programmed cell death [34,35]. Also, autophagy is essential for maintaining egg maturation cycles in *Aedes aegypti* mosquito [36].

This study showed that significant changes at the transcription level of all the identified *Aaatg* genes occurred development of *Aedes albopictus* mosquito. Also, the transcription levels of all the twelve identified *Aaatg* genes except *Aaatg5*, *Aaatg7* and *Aaatg101* were difference in male and female adult mosquitoes. This data implied that autophagy might play a role in development of *Aedes albopictus* mosquito. However, exactly how autophagy functioning in development of *Aedes albopictus* mosquito requires further investigation.

It was observed that the level of AaAtg8-II was reduced instead of increased by autophagy inducer Rapa treatment (Fig 5A). It is known that AaAtg8-II present on the inner and outer membrane of autophagosomes, with the former being degraded inside autolysosomes, and the latter cleaved by AaAtg4 and returned to the cytosol in complete autophagy [37]. Thus, the lower level of AaAtg8-II caused by Rapa treatment might be a result of a strong autophagic flux. This was proved by the fact that treatment of CQ after Rapa led to higher level of AaAtg8-II than Rapa treatment alone (Fig 5A). Also, CQ treatment alone resulted in significant increase of AaAtg8-II, indicating a basal level autophagy existence in C6/36 cells (Fig 4A).

It is known that Atg6 and Atg16 functions in initiation and elongation phase of autophagy, respectively [38]. The function of AaAtg6 and AaAtg16 were examined preliminarily. It was found that both AaAtg6 and AaAtg16 were involved in autophagy induced by Rapa, indicating they are functional Atgs (Fig 6C). Moreover, the levels of AaAtg6 and AaAtg16 changed with certain patter during SINV infection (Fig 7A and 7B). The role of autophagy in the replication of *Aedes* mosquito transmitted arbovirus including CHIKV, DENV and ZIKA virus has been reported [39–41]. SINV is a kind of arbovirus transmitted to humans through bites by *Aedes albopictus* mosquito. Therefore, our work provided preliminary information for further study of the role of autophagy in viral infection in *Aedes albopictus* mosquito.

In summary, this study provided useful information which may help the subsequent research on autophagy pathway and the role of autophagy in arboviral infection in *Aedes albopictus* mosquito.

## Supporting information

S1 Fig Sequence analysis of AaAtg1. The amino acid sequence of AaAtg1 was shown in alignment with Atg1 orthologs from *Aedes aegypti* (AeAtg1, NCBI: XP_021706125.1), *Anopheles gambiae* (AgAtg1, NCBI: XP_309350.4), *Culex quinquefasciatus* (CqAtg1, NCBI: XP_001842942.1), *Drosophila melanogaster* (DmAtg1, NCBI: NP_648601.1) and *Homo sapiens* (HsAtg1, NCBI: NP_003556.1). The alignment was performed by using ClustalX 2.1 and modified by GeneDoc 3.2. The amino acid residues identical among 6, 5 and 4 or 3 orthologs were indicated by white letters within black boxes, white letters within dark gray boxes, and black letters within light gray boxes, respectively. Secondary structures were predicted using PSIPRED 4.0. α: alpha helices; β: beta sheets.
(TIF)

S2 Fig Sequence analysis of AaAtg3. The amino acid sequence of AaAtg3 was shown in alignment with Atg3 orthologs from *Aedes aegypti* (AeAtg3, NCBI: XP_001657463.1), *Anopheles gambiae* (AgAtg3, NCBI: XP_309926.3), *Culex quinquefasciatus* (CqAtg3, NCBI: XP_001842904.1), *Drosophila melanogaster* (DmAtg3, NCBI: NP_649059.1) and *Homo sapiens* (HsAtg3, NCBI: NP_071933.2). The alignment was performed by using ClustalX 2.1 and modified by GeneDoc 3.2. The amino acid residues identical among 6, 5 and 4 or 3 orthologs were indicated by white letters within black boxes, white letters within dark gray boxes, and black letters within light gray boxes, respectively. Secondary structures were predicted using PSIPRED 4.0. α: alpha helices; β: beta sheets.
(TIF)

S3 Fig Sequence analysis of AaAtg4. The amino acid sequence of AaAtg4 was shown in alignment with Atg4 orthologs from *Aedes aegypti* (AeAtg4, NCBI: XP_001658259.2), *Anopheles gambiae* (AgAtg4, NCBI: XP_316946.4), *Culex quinquefasciatus* (CqAtg4, NCBI: XP_001846106.1), *Drosophila melanogaster* (DmAtg4, NCBI: NP_001259852.1) and *Homo sapiens* (HsAtg4A, NCBI: NP_443168.2 and HsAtg4B, NCBI: NP_037457.3). The alignment was performed by using ClustalX 2.1 and modified by GeneDoc 3.2. The amino acid residues identical among 6, 5 and 4 or 3 orthologs were indicated by white letters within black boxes, white letters within dark gray boxes, and black letters within light gray boxes, respectively. Secondary structures were predicted using PSIPRED 4.0. α: alpha helices; β: beta sheets.
(TIF)

S4 Fig Sequence analysis of AaAtg5. The amino acid sequence of AaAtg5 was shown in alignment with Atg5 orthologs from *Aedes aegypti* (AeAtg5, NCBI: XP_001661241.1), *Anopheles gambiae* (AgAtg5, NCBI: XP_309756.4), *Culex quinquefasciatus* (CqAtg5, NCBI: XP_001866028.1), *Drosophila melanogaster* (DmAtg5, NCBI: NP_572390.1) and *Homo sapiens* (HsAtg5, NCBI: NP_001273035.1). The alignment was performed by using ClustalX 2.1 and modified by GeneDoc 3.2. The amino acid residues identical among 6, 5 and 4 or 3 orthologs were indicated by white letters within black boxes, white letters within dark gray boxes, and black letters within light gray boxes, respectively. Secondary structures were predicted using PSIPRED 4.0. α: alpha helices; β: beta sheets.
(TIF)

S5 Fig Sequence analysis of AaAtg7. The amino acid sequence of AaAtg7 was shown in alignment with Atg7 orthologs from *Aedes aegypti* (AeAtg7, NCBI: XP_001660430.2), *Anopheles gambiae* (AgAtg7, NCBI: XP_318213.4), *Drosophila melanogaster* (DmAtg7, NCBI: NP_611350.1) and *Homo sapiens* (HsAtg7, NCBI: NP_001336161.1). The alignment was performed by using ClustalX 2.1 and modified by GeneDoc 3.2. The amino acid residues identical among 6, 5 and 4 or 3 orthologs were indicated by white letters within black boxes, white letters within dark gray boxes, and black letters within light gray boxes, respectively. Secondary structures were predicted using PSIPRED 4.0. α: alpha helices; β: beta sheets.
(TIF)

S6 Fig Sequence analysis of AaAtg9. The amino acid sequence of AaAtg9 was shown in alignment with Atg9 orthologs from *Aedes aegypti* (AeAtg9, NCBI: XP_021711494.1), *Anopheles gambiae* (AgAtg9, NCBI: XP_321322.5), *Culex quinquefasciatus* (CqAtg9, NCBI: XP_001863826.1), *Drosophila melanogaster* (DmAtg9, NCBI: NP_611114.1) and *Homo sapiens* (HsAtg9A, NCBI: NP_001070666.1 and HsAtg9B, NCBI: NP_001303985.1). The alignment was performed by using ClustalX 2.1 and modified by GeneDoc 3.2. The amino acid residues identical among 6, 5 and 4 or 3 orthologs were indicated by white letters within black boxes, white letters within dark gray boxes, and black letters within light gray boxes, respectively. Secondary structures were predicted using PSIPRED 4.0. α: alpha helices; β: beta sheets.
(TIF)

S7 Fig Sequence analysis of AaAtg10. The amino acid sequence of AaAtg10 was shown in alignment with Atg10 orthologs from *Aedes aegypti* (AeAtg10, NCBI: XP_021695105.1), *Anopheles gambiae* (AgAtg10, NCBI: XP_003437140.1), *Culex quinquefasciatus* (CqAtg10, NCBI: XP_001866019.1), *Drosophila melanogaster* (DmAtg10, NCBI: NP_001097216.2) and *Homo sapiens* (HsAtg10, NCBI: NP_001124500.1). The alignment was performed by using ClustalX 2.1 and modified by GeneDoc 3.2. The amino acid residues identical among 6, 5 and 4 or 3 orthologs were indicated by white letters within black boxes, white letters within dark gray boxes, and black letters within light gray boxes, respectively. Secondary structures were predicted using PSIPRED 4.0. α: alpha helices; β: beta sheets.
(TIF)

S8 Fig Sequence analysis of AaAtg12. The amino acid sequence of AaAtg12 was shown in alignment with Atg12 orthologs from *Aedes aegypti* (AeAtg12, NCBI: XP_001659704.1), *Anopheles gambiae* (AgAtg12, NCBI: XP_307342.3), *Culex quinquefasciatus* (CqAtg12, NCBI: XP_001843368.1), *Drosophila melanogaster* (DmAtg12, NCBI: NP_648551.3) and *Homo sapiens* (HsAtg12, NCBI: NP_004698.3). The alignment was performed by using ClustalX 2.1 and modified by GeneDoc 3.2. The amino acid residues identical among 6, 5 and 4 or 3 orthologs were indicated by white letters within black boxes, white letters within dark gray boxes, and black letters within light gray boxes, respectively. Secondary structures were predicted using PSIPRED 4.0. α: alpha helices; β: beta sheets.
(TIF)

S9 Fig Sequence analysis of AaAtg13. The amino acid sequence of AaAtg13 was shown in alignment with Atg13 orthologs from *Aedes aegypti* (AeAtg13, NCBI: XP_021695537.1), *Anopheles gambiae* (AgAtg13, NCBI: XP_315727.4), *Culex quinquefasciatus* (CqAtg13, NCBI: XP_001843427.1), *Drosophila melanogaster* (DmAtg13, NCBI: NP_649796.1) and *Homo sapiens* (HsAtg13, NCBI: NP_001192048.1). The alignment was performed by using ClustalX 2.1 and modified by GeneDoc 3.2. The amino acid residues identical among 6, 5 and 4 or 3 orthologs were indicated by white letters within black boxes, white letters within dark gray boxes, and black letters within light gray boxes, respectively. Secondary structures were predicted using PSIPRED 4.0. α: alpha helices; β: beta sheets.
(TIF)

S10 Fig Sequence analysis of AaAtg16. The amino acid sequence of AaAtg16 was shown in alignment with Atg16 orthologs from *Aedes aegypti* (AeAtg16, NCBI: XP_021709277.1), *Anopheles gambiae* (AgAtg16, NCBI: XP_003436086.1), *Culex quinquefasciatus* (CqAtg16, NCBI: XP_001846552.1), *Drosophila melanogaster* (DmAtg16, NCBI: NP_733313.2) and *Homo sapiens* (HsAtg16L1, NCBI: NP_060444.3 and HsAtg16L2, NCBI: NP_203746.1). The alignment was performed by using ClustalX 2.1 and modified by GeneDoc 3.2. The amino acid residues identical among 6, 5 and 4 or 3 orthologs were indicated by white letters within black boxes, white letters within dark gray boxes, and black letters within light gray boxes, respectively. Secondary structures were predicted using PSIPRED 4.0. α: alpha helices; β: beta sheets.
(TIF)

S11 Fig Sequence analysis of AaAtg101. The amino acid sequence of AaAtg101 was shown in alignment with Atg101 orthologs from *Aedes aegypti* (AeAtg101, NCBI: XP_021703515.1), *Culex quinquefasciatus* (CqAtg101, NCBI: XP_001862740.1), *Drosophila melanogaster* (DmAtg101, NCBI: NP_573326.1) and *Homo sapiens* (HsAtg101, NCBI: NP_068753.2). The alignment was performed by using ClustalX 2.1 and modified by GeneDoc 3.2. The amino acid residues identical among 6, 5 and 4 or 3 orthologs were indicated by white letters within black boxes, white letters within dark gray boxes, and black letters within light gray boxes, respectively. Secondary structures were predicted using PSIPRED 4.0. α: alpha helices; β: beta sheets.
(TIF)

S1 Table Primers used in this study. (DOCX)

S2 Table The information of siRNA used in this study. (DOCX)

S1 Raw images (PDF)
