# Supplementary figures and images for: Systematic identification of autophagy-related proteins in Aedes albopictus
Source: PLoS One. 2021 Jan 19;16(1):e0245694. doi: 10.1371/journal.pone.0245694 (PMC7815101; doi:10.1371/journal.pone.0245694)

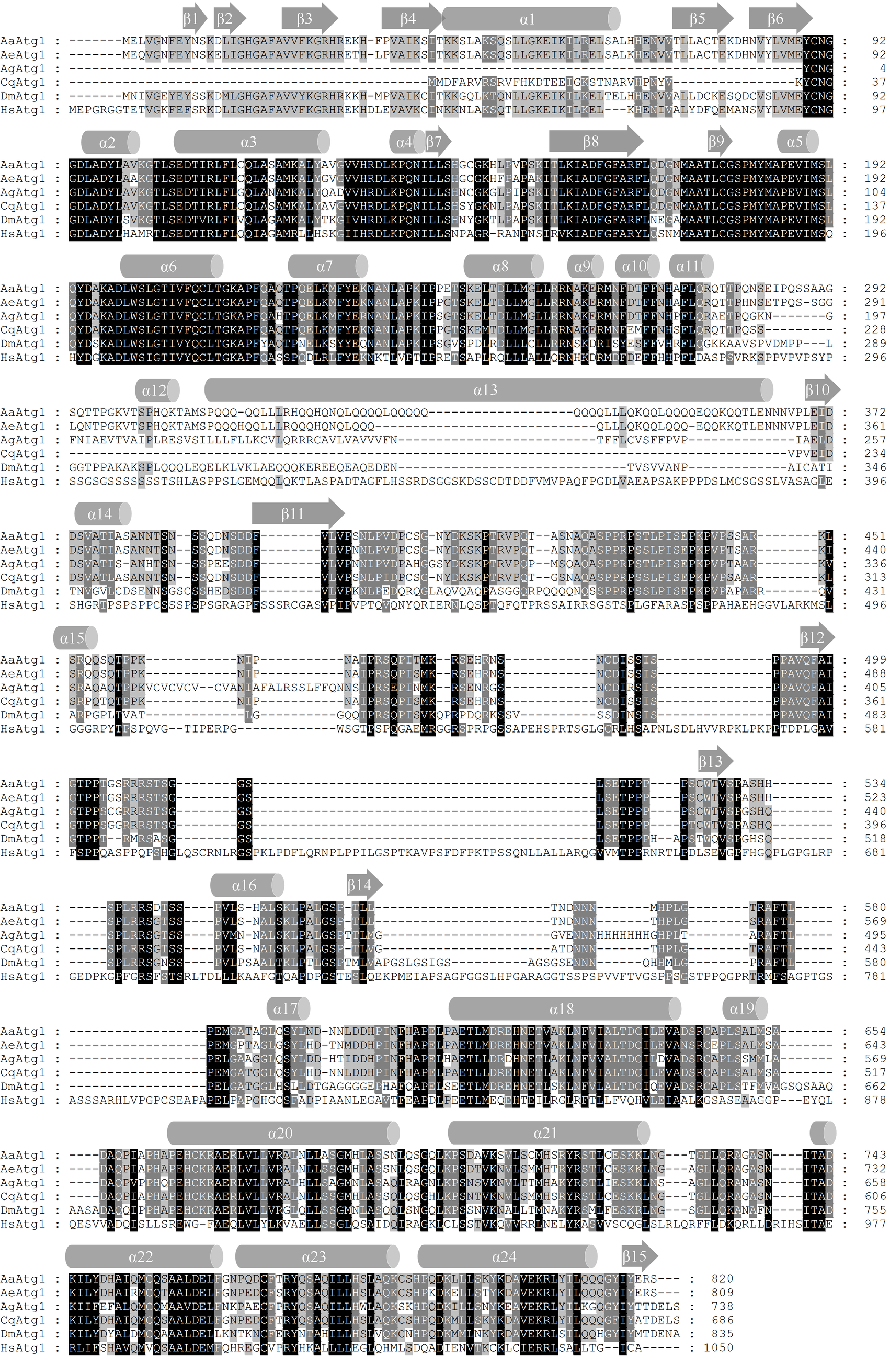

Supplement: S1 Fig — The amino acid sequence of AaAtg1 was shown in alignment with Atg1 orthologs from Aedes aegypti (AeAtg1, NCBI: XP_021706125.1), Anopheles gambiae (AgAtg1, NCBI: XP_309350.4), Culex quinquefasciatus (CqAtg1, NCBI: XP_001842942.1), Drosophila melanogaster (DmAtg1, NCBI: NP_648601.1) and Homo sapiens (HsAtg1, NCBI: NP_003556.1). The alignment was performed by using ClustalX 2.1 and modified by GeneDoc 3.2. The amino acid residues identical among 6, 5 and 4 or 3 orthologs were indicated by white letters within black boxes, white letters within dark gray boxes, and black letters within light gray boxes, respectively. Secondary structures were predicted using PSIPRED 4.0. α: alpha helices; β: beta sheets. (TIF) [file pone.0245694.s001.tif]

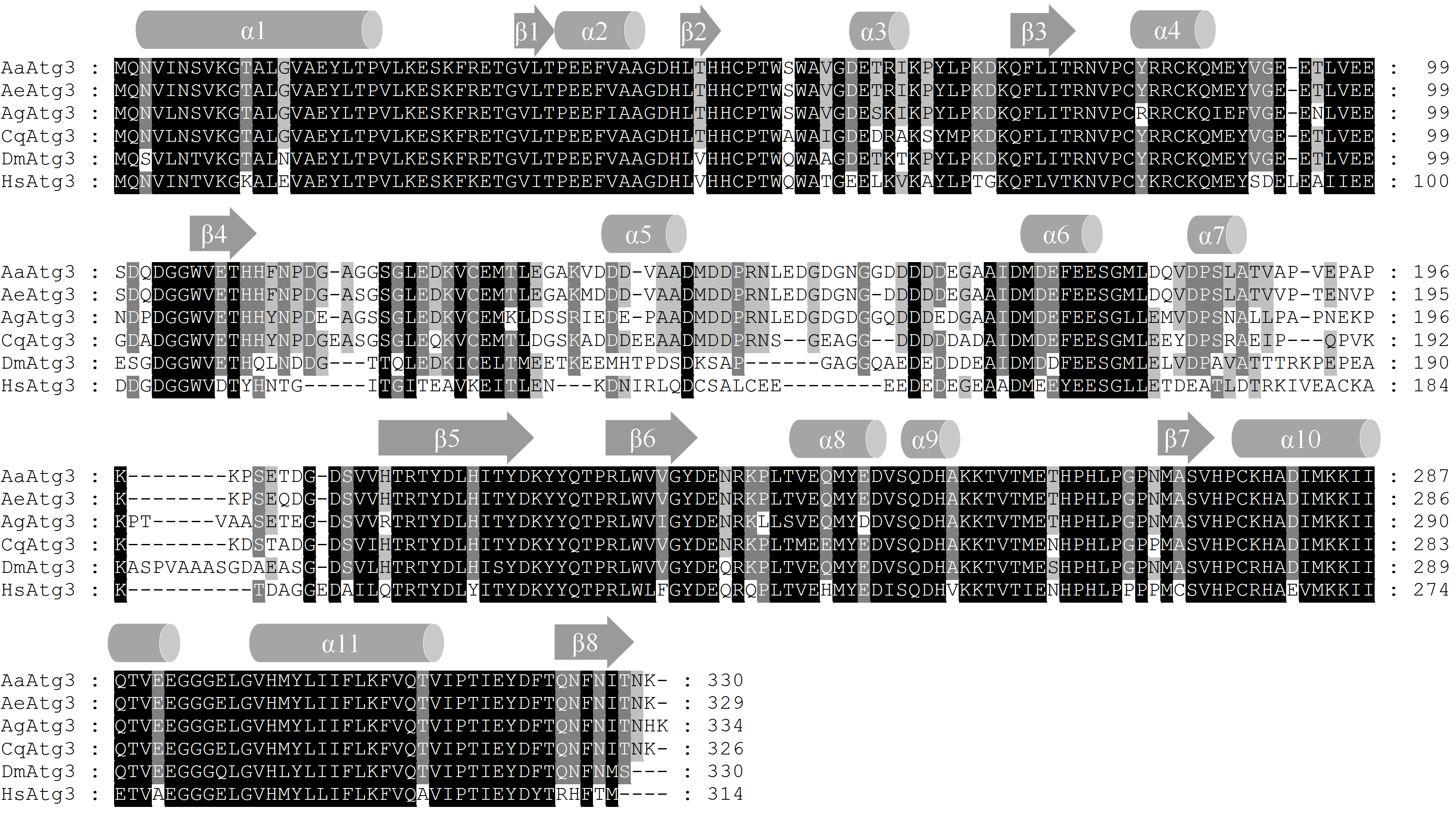

Supplement: S2 Fig — The amino acid sequence of AaAtg3 was shown in alignment with Atg3 orthologs from Aedes aegypti (AeAtg3, NCBI: XP_001657463.1), Anopheles gambiae (AgAtg3, NCBI: XP_309926.3), Culex quinquefasciatus (CqAtg3, NCBI: XP_001842904.1), Drosophila melanogaster (DmAtg3, NCBI: NP_649059.1) and Homo sapiens (HsAtg3, NCBI: NP_071933.2). The alignment was performed by using ClustalX 2.1 and modified by GeneDoc 3.2. The amino acid residues identical among 6, 5 and 4 or 3 orthologs were indicated by white letters within black boxes, white letters within dark gray boxes, and black letters within light gray boxes, respectively. Secondary structures were predicted using PSIPRED 4.0. α: alpha helices; β: beta sheets. (TIF) [file pone.0245694.s002.tif]

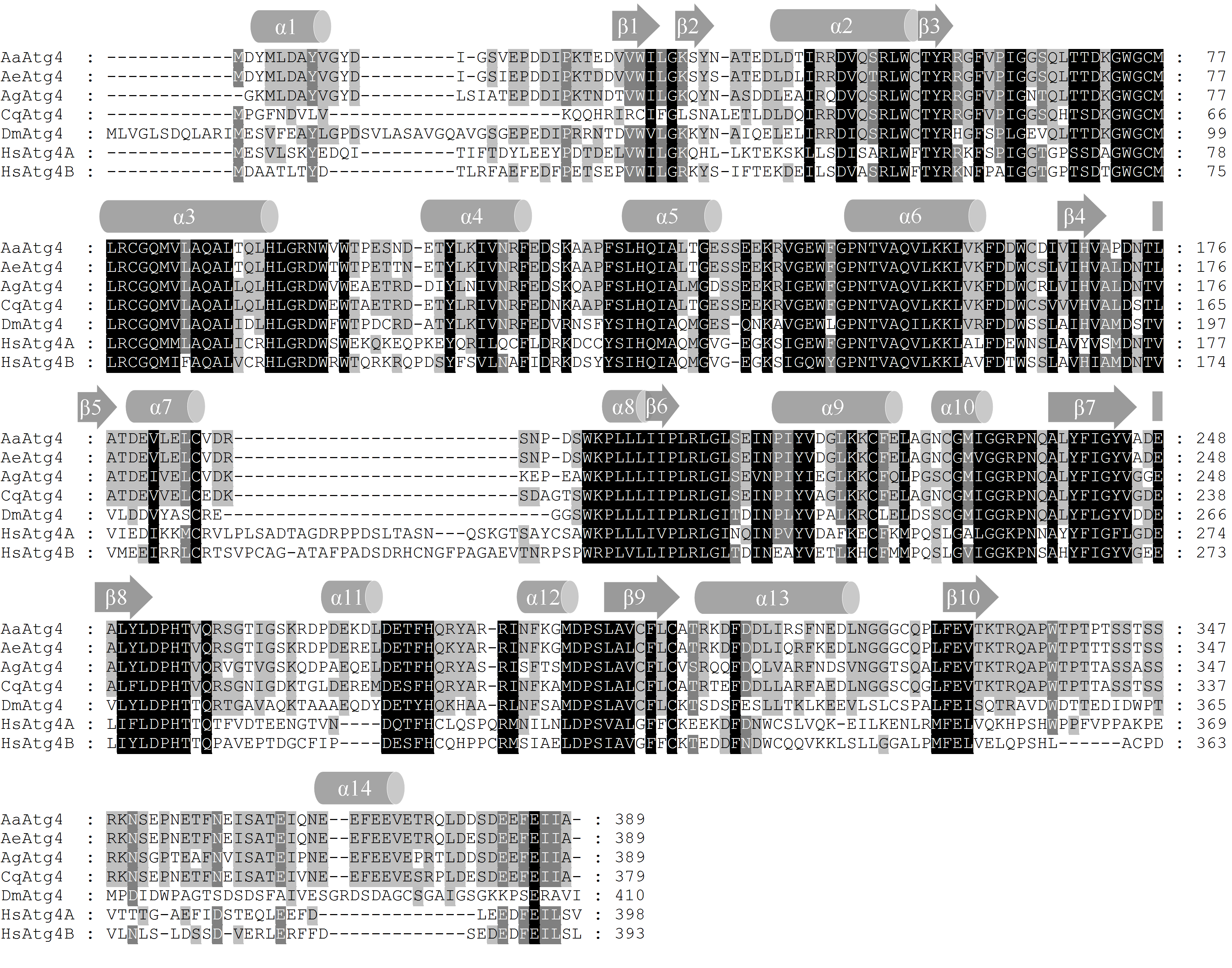

Supplement: S3 Fig — The amino acid sequence of AaAtg4 was shown in alignment with Atg4 orthologs from Aedes aegypti (AeAtg4, NCBI: XP_001658259.2), Anopheles gambiae (AgAtg4, NCBI: XP_316946.4), Culex quinquefasciatus (CqAtg4, NCBI: XP_001846106.1), Drosophila melanogaster (DmAtg4, NCBI: NP_001259852.1) and Homo sapiens (HsAtg4A, NCBI: NP_443168.2 and HsAtg4B, NCBI: NP_037457.3). The alignment was performed by using ClustalX 2.1 and modified by GeneDoc 3.2. The amino acid residues identical among 6, 5 and 4 or 3 orthologs were indicated by white letters within black boxes, white letters within dark gray boxes, and black letters within light gray boxes, respectively. Secondary structures were predicted using PSIPRED 4.0. α: alpha helices; β: beta sheets. (TIF) [file pone.0245694.s003.tif]

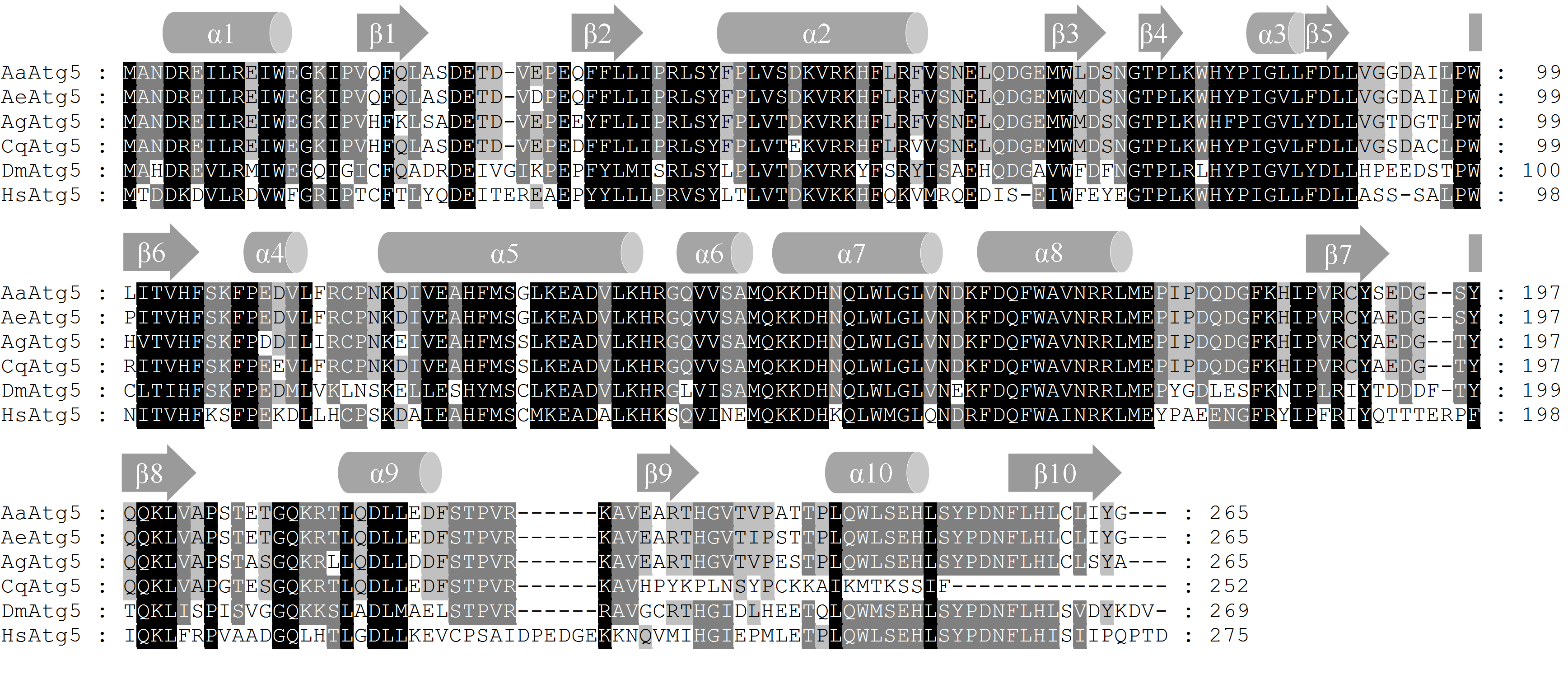

Supplement: S4 Fig — The amino acid sequence of AaAtg5 was shown in alignment with Atg5 orthologs from Aedes aegypti (AeAtg5, NCBI: XP_001661241.1), Anopheles gambiae (AgAtg5, NCBI: XP_309756.4), Culex quinquefasciatus (CqAtg5, NCBI: XP_001866028.1), Drosophila melanogaster (DmAtg5, NCBI: NP_572390.1) and Homo sapiens (HsAtg5, NCBI: NP_001273035.1). The alignment was performed by using ClustalX 2.1 and modified by GeneDoc 3.2. The amino acid residues identical among 6, 5 and 4 or 3 orthologs were indicated by white letters within black boxes, white letters within dark gray boxes, and black letters within light gray boxes, respectively. Secondary structures were predicted using PSIPRED 4.0. α: alpha helices; β: beta sheets. (TIF) [file pone.0245694.s004.tif]

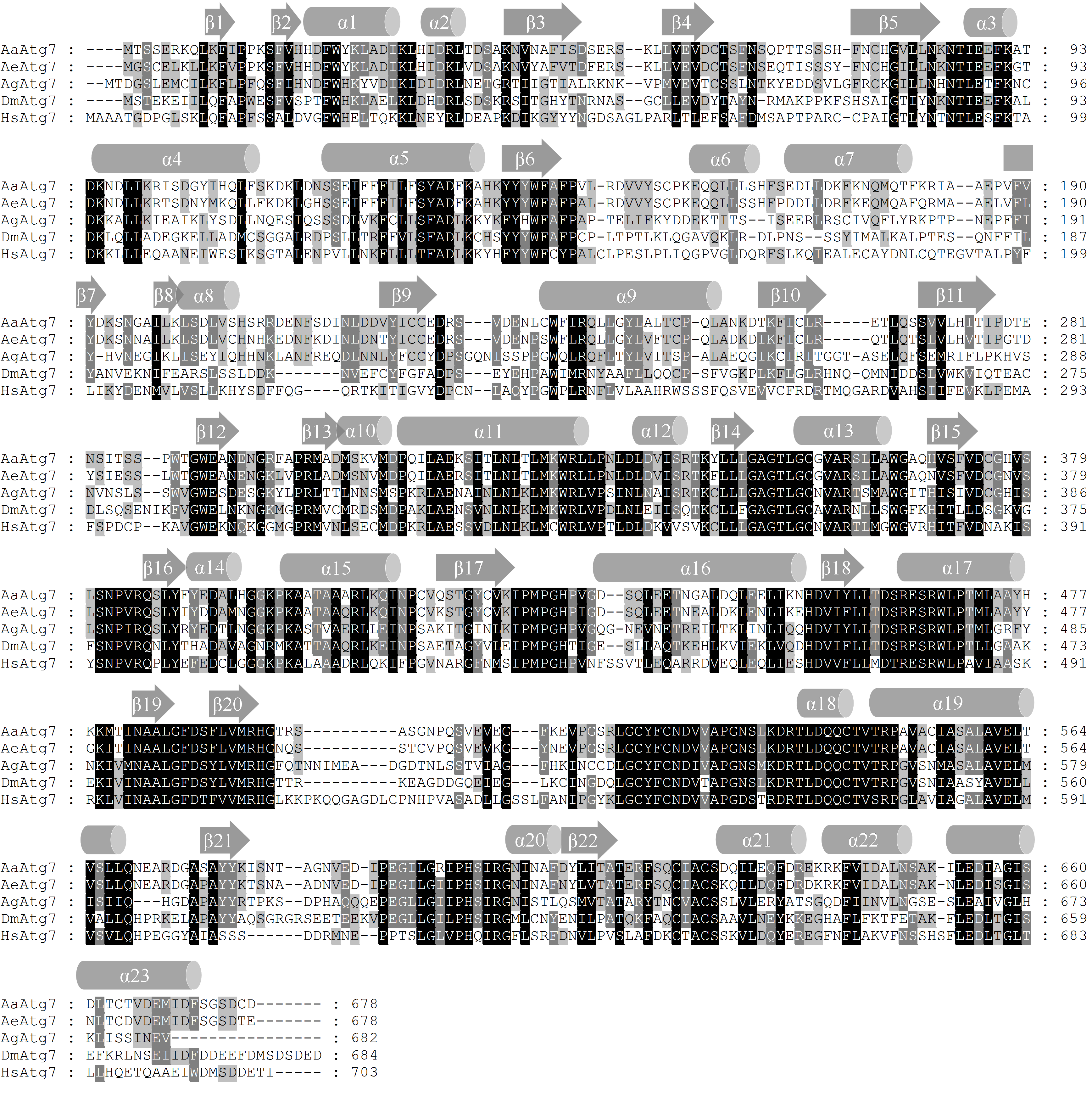

Supplement: S5 Fig — The amino acid sequence of AaAtg7 was shown in alignment with Atg7 orthologs from Aedes aegypti (AeAtg7, NCBI: XP_001660430.2), Anopheles gambiae (AgAtg7, NCBI: XP_318213.4), Drosophila melanogaster (DmAtg7, NCBI: NP_611350.1) and Homo sapiens (HsAtg7, NCBI: NP_001336161.1). The alignment was performed by using ClustalX 2.1 and modified by GeneDoc 3.2. The amino acid residues identical among 6, 5 and 4 or 3 orthologs were indicated by white letters within black boxes, white letters within dark gray boxes, and black letters within light gray boxes, respectively. Secondary structures were predicted using PSIPRED 4.0. α: alpha helices; β: beta sheets. (TIF) [file pone.0245694.s005.tif]

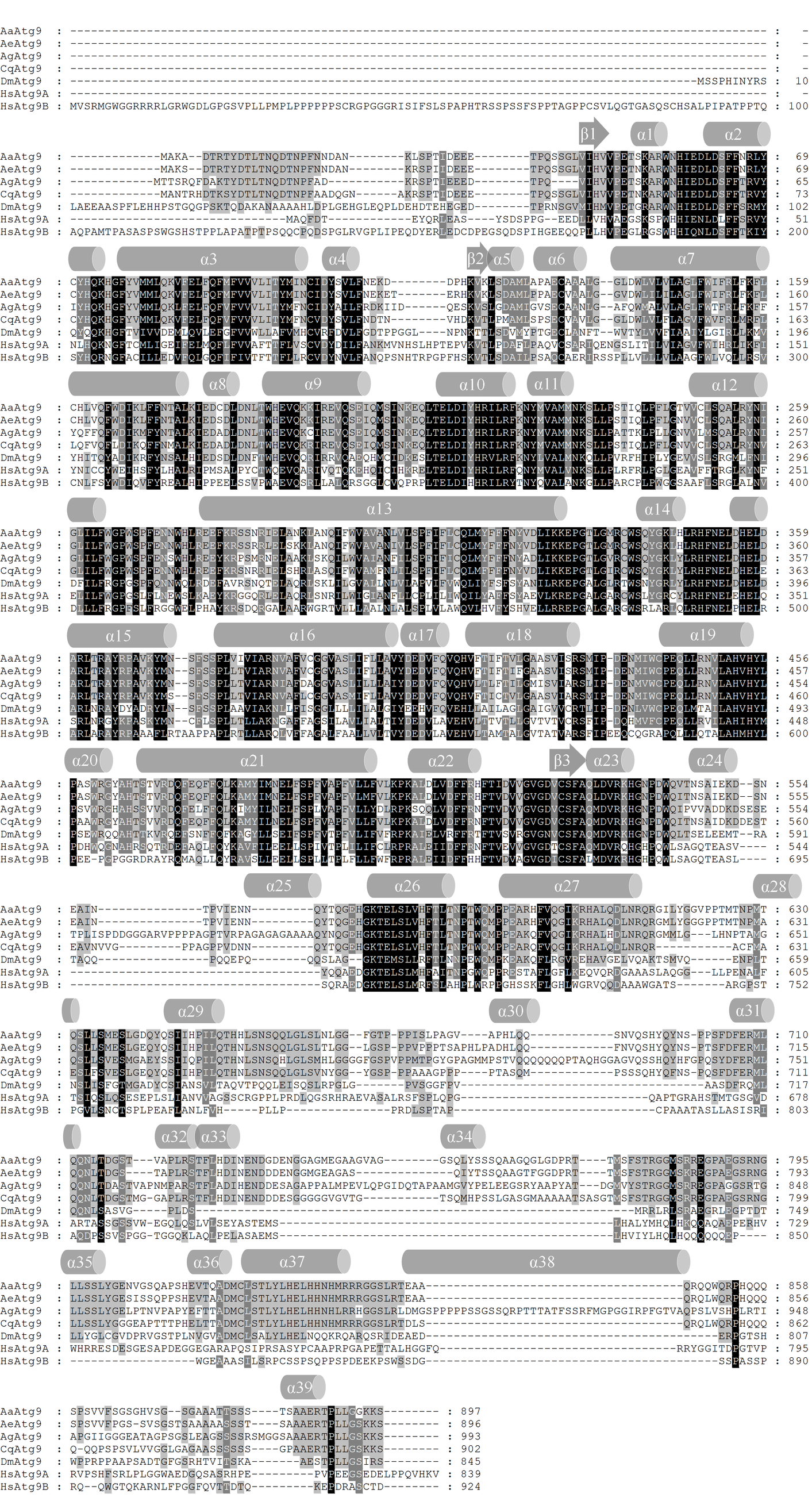

Supplement: S6 Fig — The amino acid sequence of AaAtg9 was shown in alignment with Atg9 orthologs from Aedes aegypti (AeAtg9, NCBI: XP_021711494.1), Anopheles gambiae (AgAtg9, NCBI: XP_321322.5), Culex quinquefasciatus (CqAtg9, NCBI: XP_001863826.1), Drosophila melanogaster (DmAtg9, NCBI: NP_611114.1) and Homo sapiens (HsAtg9A, NCBI: NP_001070666.1 and HsAtg9B, NCBI: NP_001303985.1). The alignment was performed by using ClustalX 2.1 and modified by GeneDoc 3.2. The amino acid residues identical among 6, 5 and 4 or 3 orthologs were indicated by white letters within black boxes, white letters within dark gray boxes, and black letters within light gray boxes, respectively. Secondary structures were predicted using PSIPRED 4.0. α: alpha helices; β: beta sheets. (TIF) [file pone.0245694.s006.tif]

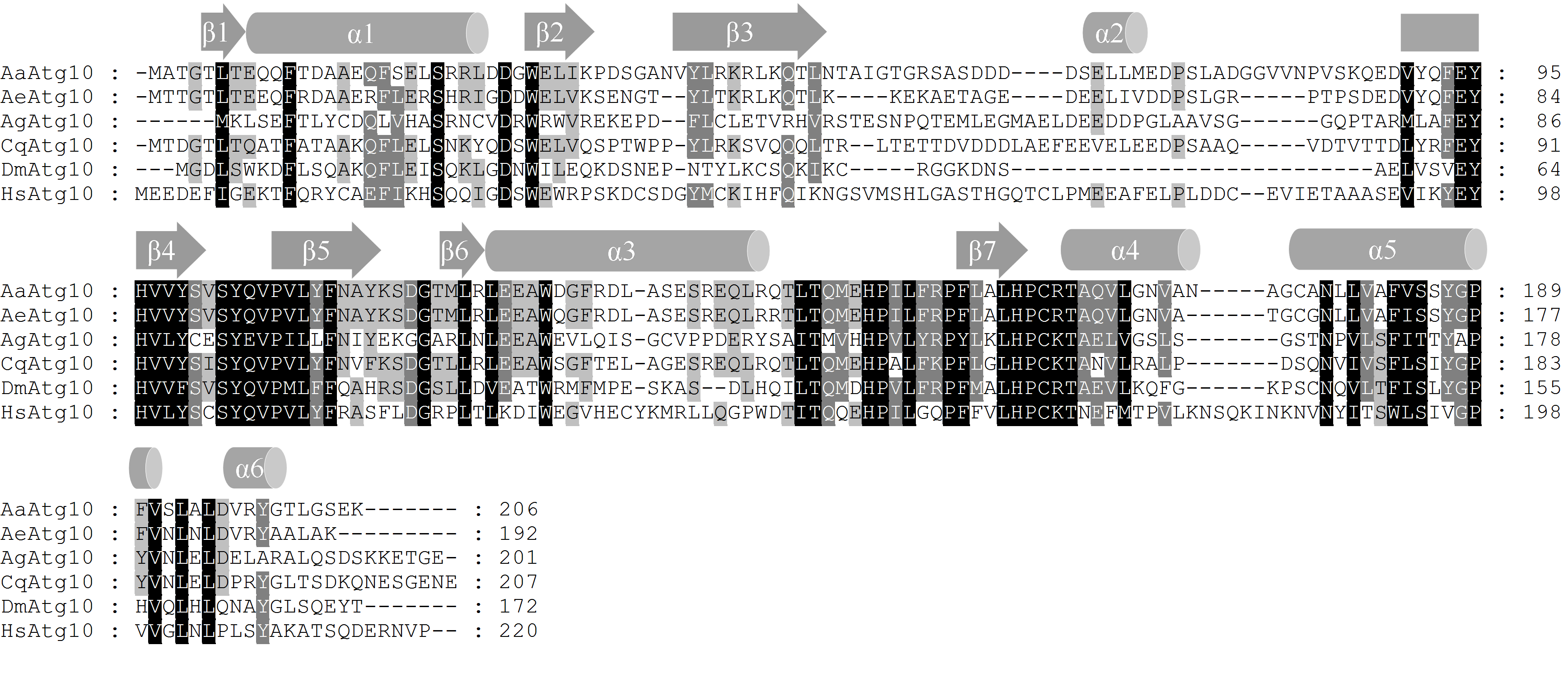

Supplement: S7 Fig — The amino acid sequence of AaAtg10 was shown in alignment with Atg10 orthologs from Aedes aegypti (AeAtg10, NCBI: XP_021695105.1), Anopheles gambiae (AgAtg10, NCBI: XP_003437140.1), Culex quinquefasciatus (CqAtg10, NCBI: XP_001866019.1), Drosophila melanogaster (DmAtg10, NCBI: NP_001097216.2) and Homo sapiens (HsAtg10, NCBI: NP_001124500.1). The alignment was performed by using ClustalX 2.1 and modified by GeneDoc 3.2. The amino acid residues identical among 6, 5 and 4 or 3 orthologs were indicated by white letters within black boxes, white letters within dark gray boxes, and black letters within light gray boxes, respectively. Secondary structures were predicted using PSIPRED 4.0. α: alpha helices; β: beta sheets. (TIF) [file pone.0245694.s007.tif]

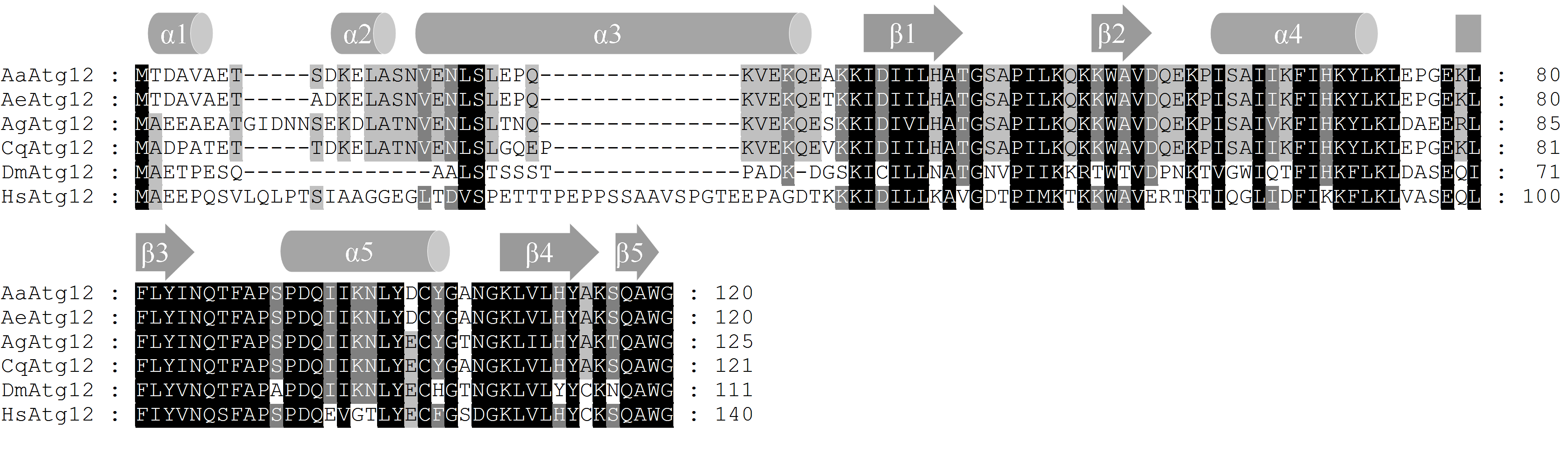

Supplement: S8 Fig — The amino acid sequence of AaAtg12 was shown in alignment with Atg12 orthologs from Aedes aegypti (AeAtg12, NCBI: XP_001659704.1), Anopheles gambiae (AgAtg12, NCBI: XP_307342.3), Culex quinquefasciatus (CqAtg12, NCBI: XP_001843368.1), Drosophila melanogaster (DmAtg12, NCBI: NP_648551.3) and Homo sapiens (HsAtg12, NCBI: NP_004698.3). The alignment was performed by using ClustalX 2.1 and modified by GeneDoc 3.2. The amino acid residues identical among 6, 5 and 4 or 3 orthologs were indicated by white letters within black boxes, white letters within dark gray boxes, and black letters within light gray boxes, respectively. Secondary structures were predicted using PSIPRED 4.0. α: alpha helices; β: beta sheets. (TIF) [file pone.0245694.s008.tif]

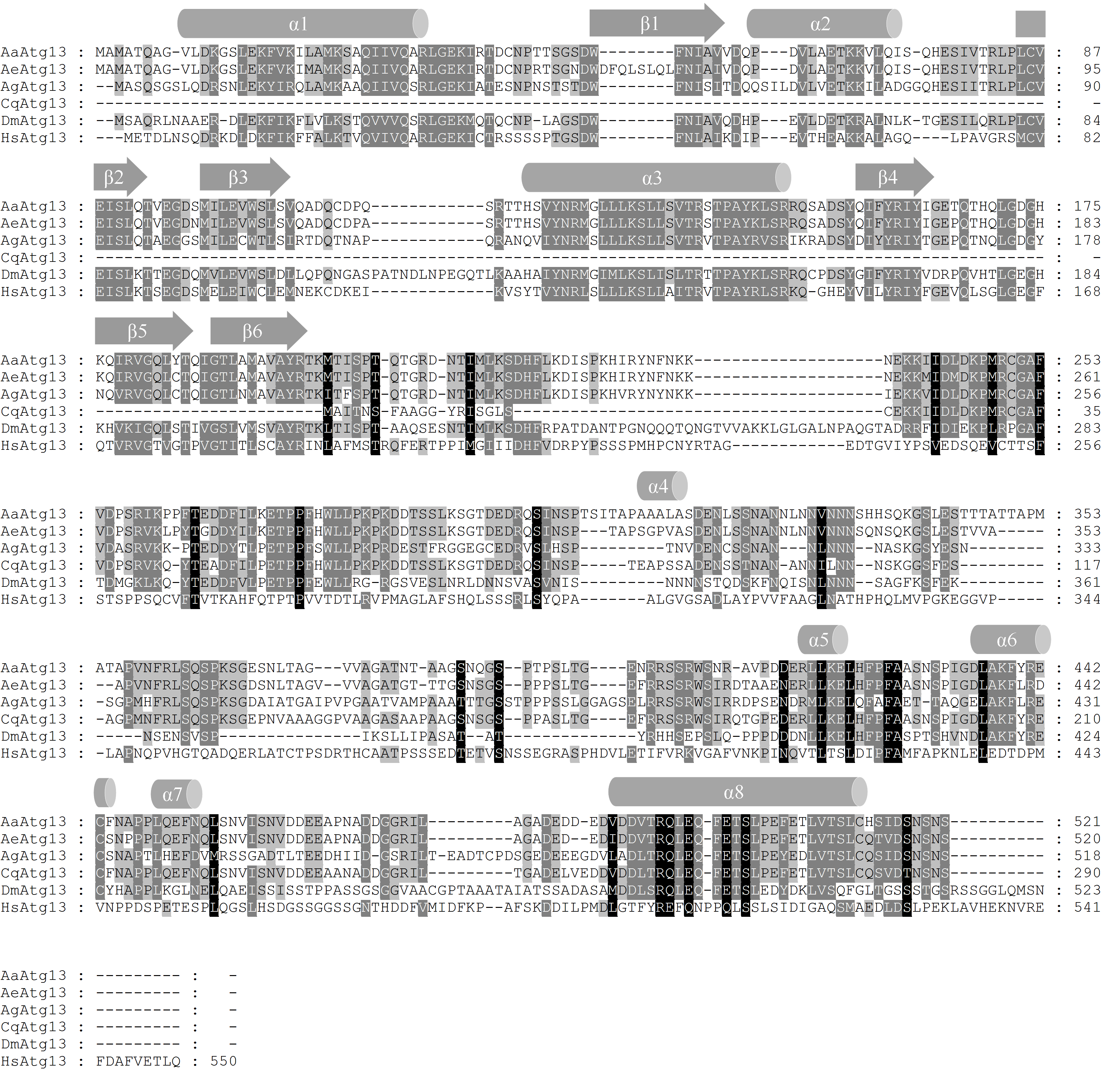

Supplement: S9 Fig — The amino acid sequence of AaAtg13 was shown in alignment with Atg13 orthologs from Aedes aegypti (AeAtg13, NCBI: XP_021695537.1), Anopheles gambiae (AgAtg13, NCBI: XP_315727.4), Culex quinquefasciatus (CqAtg13, NCBI: XP_001843427.1), Drosophila melanogaster (DmAtg13, NCBI: NP_649796.1) and Homo sapiens (HsAtg13, NCBI: NP_001192048.1). The alignment was performed by using ClustalX 2.1 and modified by GeneDoc 3.2. The amino acid residues identical among 6, 5 and 4 or 3 orthologs were indicated by white letters within black boxes, white letters within dark gray boxes, and black letters within light gray boxes, respectively. Secondary structures were predicted using PSIPRED 4.0. α: alpha helices; β: beta sheets. (TIF) [file pone.0245694.s009.tif]

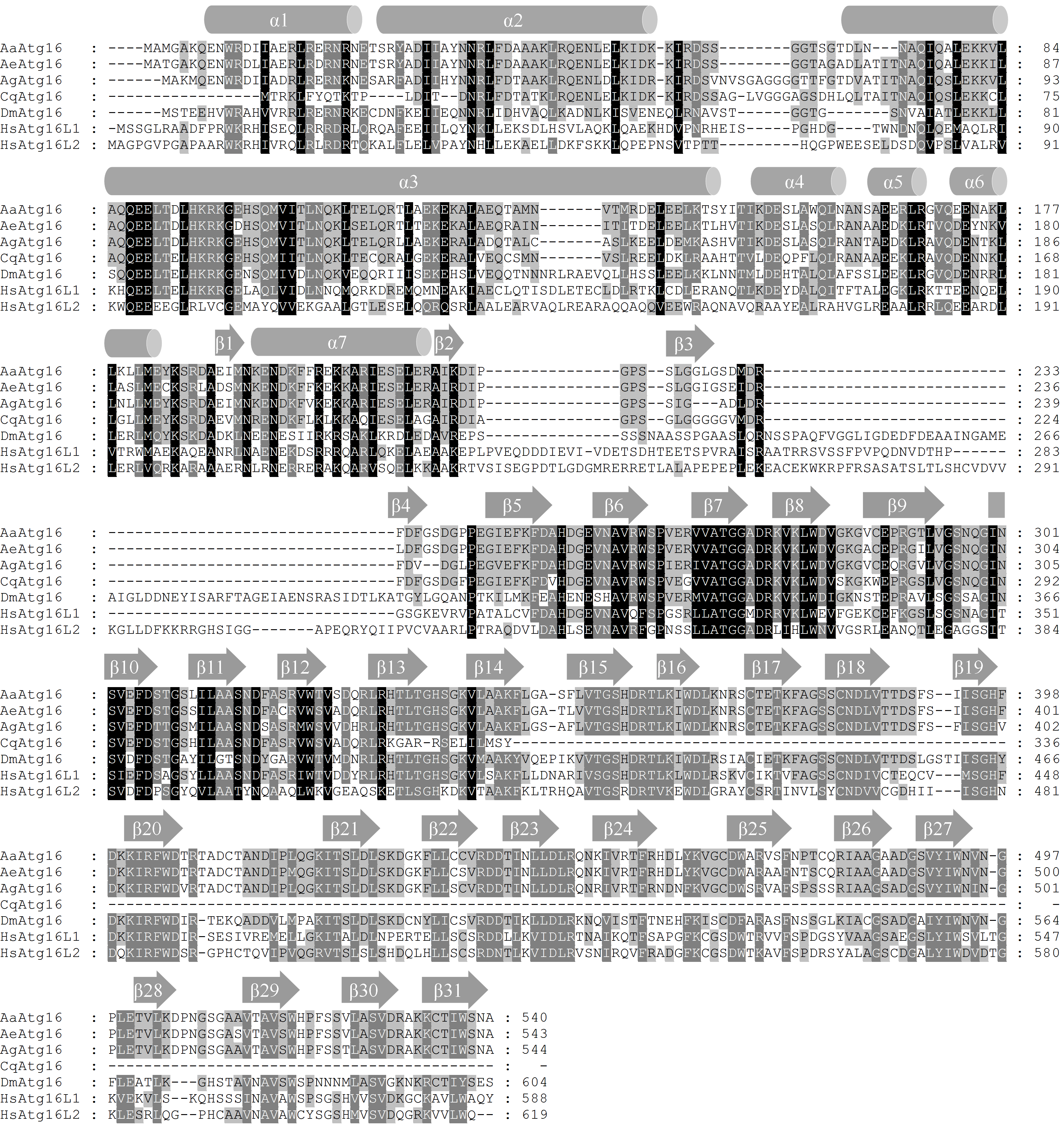

Supplement: S10 Fig — The amino acid sequence of AaAtg16 was shown in alignment with Atg16 orthologs from Aedes aegypti (AeAtg16, NCBI: XP_021709277.1), Anopheles gambiae (AgAtg16, NCBI: XP_003436086.1), Culex quinquefasciatus (CqAtg16, NCBI: XP_001846552.1), Drosophila melanogaster (DmAtg16, NCBI: NP_733313.2) and Homo sapiens (HsAtg16L1, NCBI: NP_060444.3 and HsAtg16L2, NCBI: NP_203746.1). The alignment was performed by using ClustalX 2.1 and modified by GeneDoc 3.2. The amino acid residues identical among 6, 5 and 4 or 3 orthologs were indicated by white letters within black boxes, white letters within dark gray boxes, and black letters within light gray boxes, respectively. Secondary structures were predicted using PSIPRED 4.0. α: alpha helices; β: beta sheets. (TIF) [file pone.0245694.s010.tif]

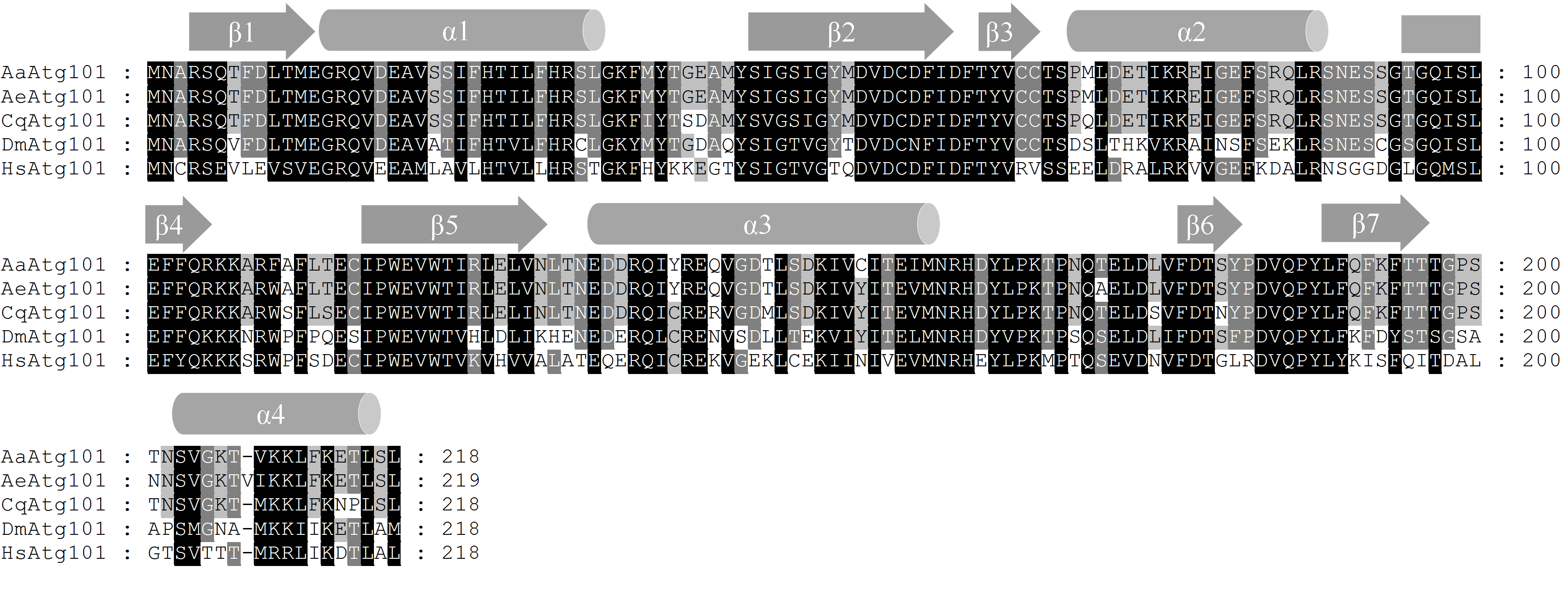

Supplement: S11 Fig — The amino acid sequence of AaAtg101 was shown in alignment with Atg101 orthologs from Aedes aegypti (AeAtg101, NCBI: XP_021703515.1), Culex quinquefasciatus (CqAtg101, NCBI: XP_001862740.1), Drosophila melanogaster (DmAtg101, NCBI: NP_573326.1) and Homo sapiens (HsAtg101, NCBI: NP_068753.2). The alignment was performed by using ClustalX 2.1 and modified by GeneDoc 3.2. The amino acid residues identical among 6, 5 and 4 or 3 orthologs were indicated by white letters within black boxes, white letters within dark gray boxes, and black letters within light gray boxes, respectively. Secondary structures were predicted using PSIPRED 4.0. α: alpha helices; β: beta sheets. (TIF) [file pone.0245694.s011.tif]
