# Supplementary material for: Systematic identification of autophagy-related proteins in Aedes albopictus
Source: PLoS One. 2021 Jan 19;16(1):e0245694. doi: 10.1371/journal.pone.0245694 (PMC7815101; doi:10.1371/journal.pone.0245694)
Supplement: S1 Table — (DOCX) [file pone.0245694.s012.docx]

**S1 Table.** **Primers used in this study**

| **Primer name** | **Description** | **Primer sequence(5’-3’)** |
| --- | --- | --- |
| AaAtg1-3’RACE  AaAtg1-5’RACE  AaAtg1-F  AaAtg1-R  AaAtg1-RT-F  AaAtg1-RT-R  AaAtg3-3’RACE  AaAtg3-5’RACE  AaAtg3-F  AaAtg3-R  AaAtg3-RT-F  AaAtg3-RT-R  AaAtg4-3’RACE  AaAtg4-5’RACE  AaAtg4-F  AaAtg4-R  AaAtg4-RT-F  AaAtg4-RT-R  AaAtg5-3’RACE  AaAtg5-5’RACE  AaAtg5-F  AaAtg5-R  AaAtg5-RT-F  AaAtg5-RT-R  AaAtg6-3’RACE  AaAtg6-5’RACE  AaAtg6-F  AaAtg6-R  AaAtg6-RT-F  AaAtg6-RT-R  AaAtg7-3’RACE  AaAtg7-5’RACE  AaAtg7-F  AaAtg7-R  AaAtg7-RT-F  AaAtg7-RT-R  AaAtg9-3’RACE  AaAtg9-5’RACE  AaAtg9-F  AaAtg9-R  AaAtg9-RT-F  AaAtg9-RT-R  AaAtg10-3’RACE  AaAtg10-5’RACE  AaAtg10-F  AaAtg10-R  AaAtg10-RT-F  AaAtg10-RT-R  AaAtg12-3’RACE  AaAtg12-5’RACE  AaAtg12-F  AaAtg12-R  AaAtg12-RT-F  AaAtg12-RT-R  AaAtg13-3’RACE  AaAtg13-5’RACE  AaAtg13-F  AaAtg13-R  AaAtg13-RT-F  AaAtg13-RT-R  AaAtg16-3’RACE  AaAtg16-5’RACE  AaAtg16-F  AaAtg16-R  AaAtg16-RT-F  AaAtg16-RT-R  AaAtg101-3’RACE  AaAtg101-5’RACE  AaAtg101-F  AaAtg101-R  AaAtg101-RT-F  AaAtg101-RT-R  AaS7-341-F  AaS7-488-R | 3’RACE primer  5’RACE primer  PCR primer, forward  PCR primer, reverse  Real-time PCR primer, forward  Real-time PCR primer, reverse  3’RACE primer  5’RACE primer  PCR primer, forward  PCR primer, revers  Real-time PCR primer, forward  Real-time PCR primer, reverse  3’RACE primer  5’RACE primer  PCR primer, forward  PCR primer, reverse  Real-time PCR primer, forward  Real-time PCR primer, reverse  3’RACE primer  5’RACE primer  PCR primer, forward  PCR primer, reverse  Real-time PCR primer, forward  Real-time PCR primer, reverse  3’RACE primer  5’RACE primer  PCR primer, forward  PCR primer, reverse  Real-time PCR primer, forward  Real-time PCR primer, reverse  3’RACE primer  5’RACE primer  PCR primer, forward  PCR primer, reverse  Real-time PCR primer, forward  Real-time PCR primer, reverse  3’RACE primer  5’RACE prime  PCR primer, forward  PCR primer, reverse  Real-time PCR primer, forward  Real-time PCR primer, reverse  3’RACE primer  5’RACE primer  PCR primer, forward  PCR primer, reverse  Real-time PCR primer, forward  Real-time PCR primer, reverse  3’RACE primer  5’RACE primer  PCR primer, forward  PCR primer, reverse  Real-time PCR primer, forward  Real-time PCR primer, reverse  3’RACE primer  5’RACE primer  PCR primer, forward  PCR primer, reverse  Real-time PCR primer, forward  Real-time PCR primer, reverse  3’RACE primer  5’RACE primer  PCR primer, forward  PCR primer, reverse  Real-time PCR primer, forward  Real-time PCR primer, reverse  3’RACE primer  5’RACE primer  PCR primer, forward  PCR primer, reverse  Real-time PCR primer, forward  Real-time PCR primer, reverse  Real-time PCR primer, forward  Real-time PCR primer, reverse | GCAGTCAGGACAATTCGGAC  CTAGGTCGCGGTGGACTCG  ATGGAACTGGTTGGGAACTTT  TCAAGACCGCTCGTAGATGTA  GCTGAATTTCGTCATCGCCC  CCCGGATGCGAGTAGATTCA  GGGAGCCAAAATGGATGATG  AGGTGATGTGCAGATCGTAGG  ATGCAAAACGTGATCAACTCG  TTACTTATTGGTAATGTTGAAATTT  TACGTCGGGGAGGAAACTCT  GTCCCCGTCTTCCAGATTCC  CGGCGAATGGTTCGGACC  CGCTAAGCCCCAGTCGC  ATGGATTACATGTTGGATGCA  TTACGCGATGATTTCGAACTC  CTACGGAAGACCTCGACACG  TGCCAGAACCATCTGACCAC  GAGGACGTGCTGTTTCGGT  CCAGAATTGGTCAAACTTGTC  ATGGCAAACGATCGGGAAA  TTAACCGTAGATCAAGCAGAGAT  TCGGCGACTAATGGAGCCTA  GGACAGGTGTCGAGAAGTCC  GTGGAGAAGCAGAGGCTTGG  TGAACACGTTGGTTTTCTTTAG  ATGAACGACGCCAAAGTCAG  TCAAACGGCTTGTTTCTCTTC  GGCACCATCAACAACTTCCG  CCGTCGCTCAGCACCTCTAT  GGGAAGCAAACGAGAATGG  GACCGAGCAACTCCACAGC  ATGACATCCAGTGAACGGAAG  TTAGTCACAATCACTACCGCTGA  CCAGTGAACGGAAGCAGCTA  GGCTGAATCGGTCAGCCTAT  GAAATGTGGCTTTCGTTTGC  GAACTGATCGCGAACTGTCG  ATGGCTAAGGCCGATACGA  CTAGGACTTTTTGCCCCCG  GCCCAGTTGGATGTCCGTAA  TCTGCCACGTGGGATTAGTT  GAAGATGTCTATCAGTTCGAGTATC  CCAGGCTTCCTCCAGTCG  ATGGCCACCGGTACCCTA  TTATTTCTCGCTACCCAATGTG  AGCCAACGTGTACCTACGAA  TTCACCACTCCTCCATCCG  GAACCACAGAAAGTGGAAAAAC  GTTTGGTTTATGTATAAGAACAATTT  ATGACTGACGCTGTTGCGG  TCATCCCCAGGCTTGACTC  CAAACATTCGCCCCATCTCC  TCATCCCCAGGCTTGACTCT  GATATCAGCCCCAAGCACATC  GTCCTTGGGCTTCGGGA  ATGGCGATGGCGACCCAG  CTAACTGTTGGAATTGCTATCGA  ATCCTCCAATGCGAACAACCTG  CTTGGGTGACTGTGATAGCCG  CGATTTTGGTTCCGATGG  CTGTCAAATTCCACAGAATTTATAC  ATGGCCATGGGTGCGAAG  CTAAGCGTTAGACCAAATTGTGC  AAGTGAACTGGAACGAGCAATC  CTTTACTTTCCTATCAGCACCACC  CGCAGCAATGAAAGCTCTG  CTTGTTCCCGATAAATCTGC  ATGAATGCTCGCTCGCAAA  TCACAACGAGAGCGTTTCCTT  ATGGACGTGGACTGCGATTT  TAGAAAAGCGAACCGTGCCT  GTCCACGATCCCGCACTCT  GTGGTCTGCTGGTTCTTGTCC |
