# Supplementary material for: Systematic identification of autophagy-related proteins in Aedes albopictus
Source: PLoS One. 2021 Jan 19;16(1):e0245694. doi: 10.1371/journal.pone.0245694 (PMC7815101; doi:10.1371/journal.pone.0245694)
Supplement: S2 Table — (DOCX) [file pone.0245694.s013.docx]

**S2 Table. The information of siRNA used in this study**

| **siRNA Description** | **Sequence information** |
| --- | --- |
| siAaAtg6-1 anti-sense | UUCGUAUCCCAGAAGAACCGA |
| siAaAtg6-1 sense | GGUUCUUCUGGGAUACGAAAU |
| siAaAtg6-2 anti-sense | UUCAAUGUCCACUUCGAUGUG |
| siAaAtg6-2 sense | CAUCGAAGUGGACAUUGAAUC |
| siAaAtg16-1 anti-sense | UAAUGUAUGAAGUCUUAAGCU |
| siAaAtg16-1 sense | CUUAAGACUUCAUACAUUACA |
| siAaAtg16-2 anti-sense | AUUUGAAGCAGCUAGAAUCAA |
| siAaAtg16-2 sense | GAUUCUAGCUGCUUCAAAUGA |
