# Supplementary material for: Systematic identification of autophagy-related proteins in Aedes albopictus
Source: PLoS One. 2021 Jan 19;16(1):e0245694. doi: 10.1371/journal.pone.0245694 (PMC7815101; doi:10.1371/journal.pone.0245694)
Supplement: S1 Raw images — (PDF) [file pone.0245694.s014.pdf]

Fig 4A

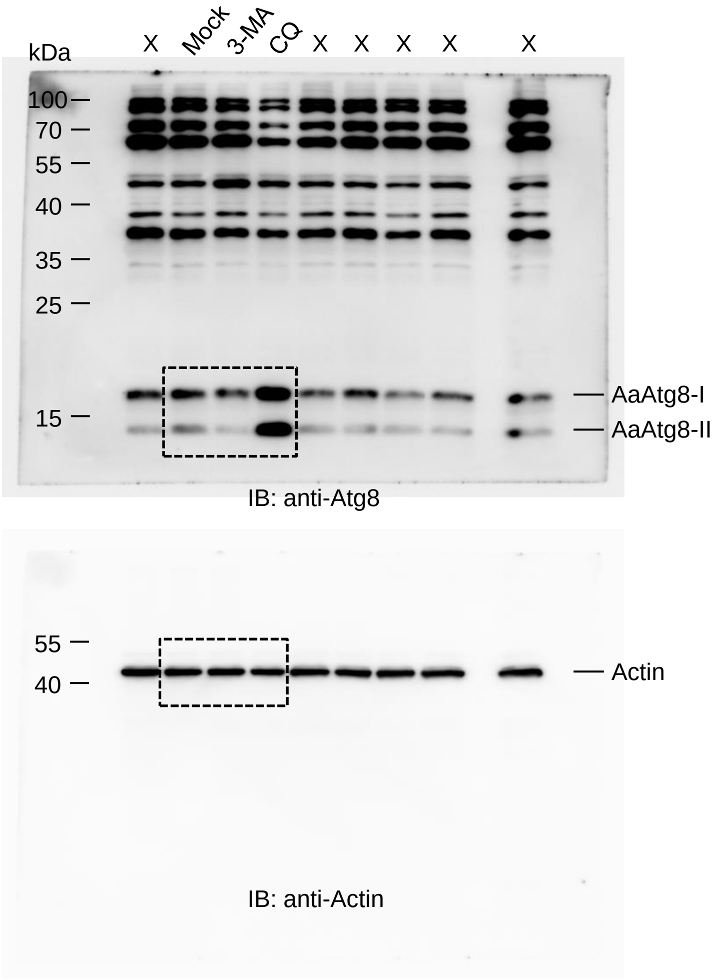

Fig 4B

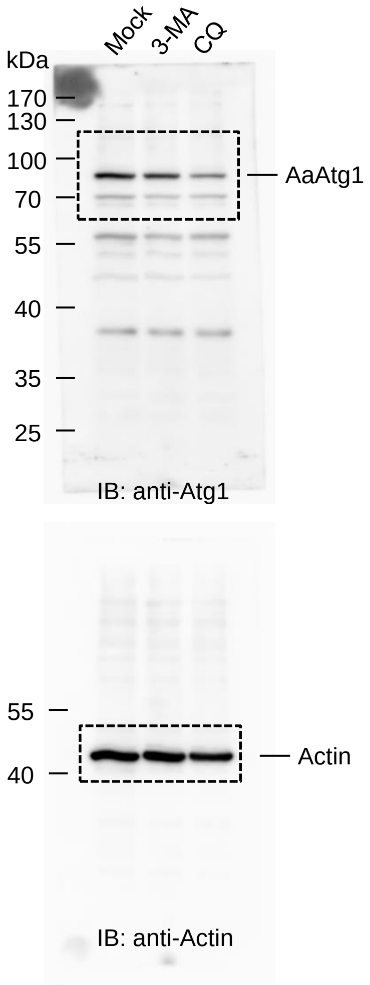

Fig 4C

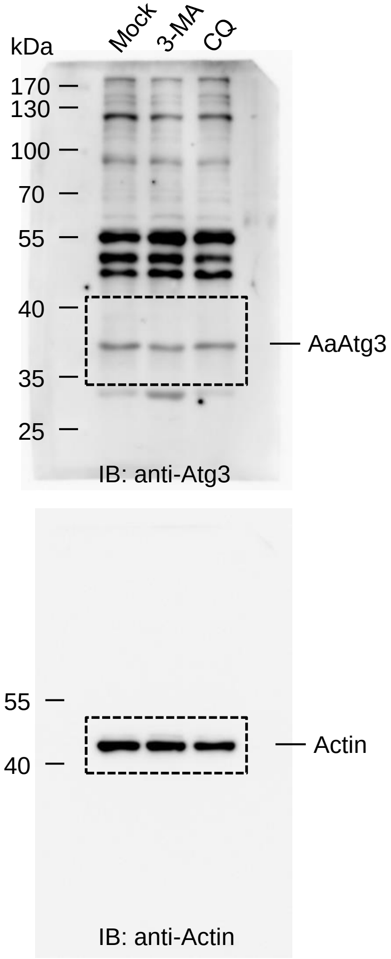

Fig 4D

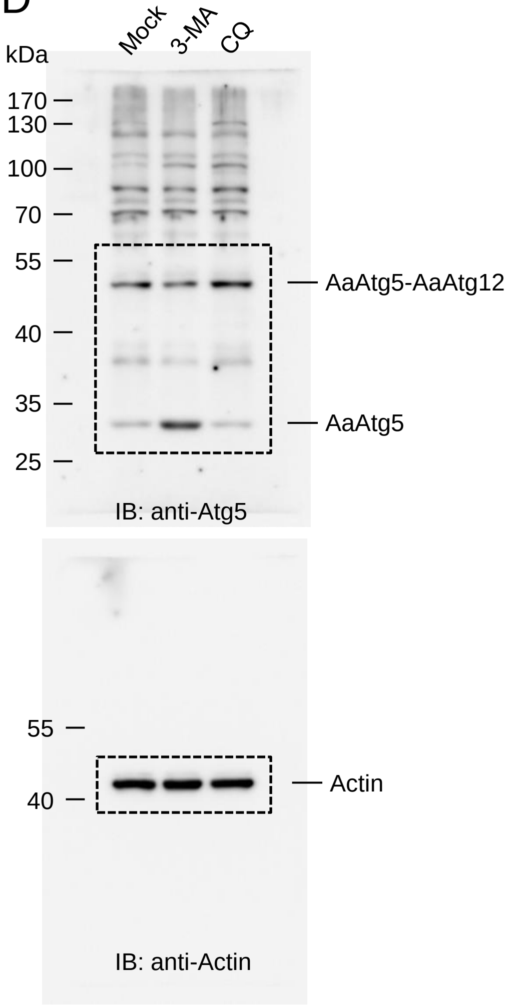

Fig 4E

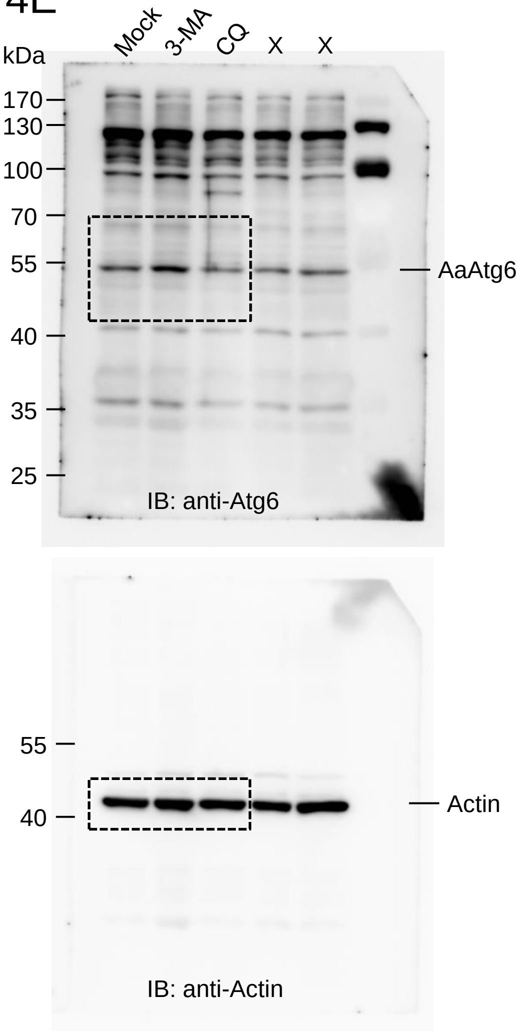

Fig 4F

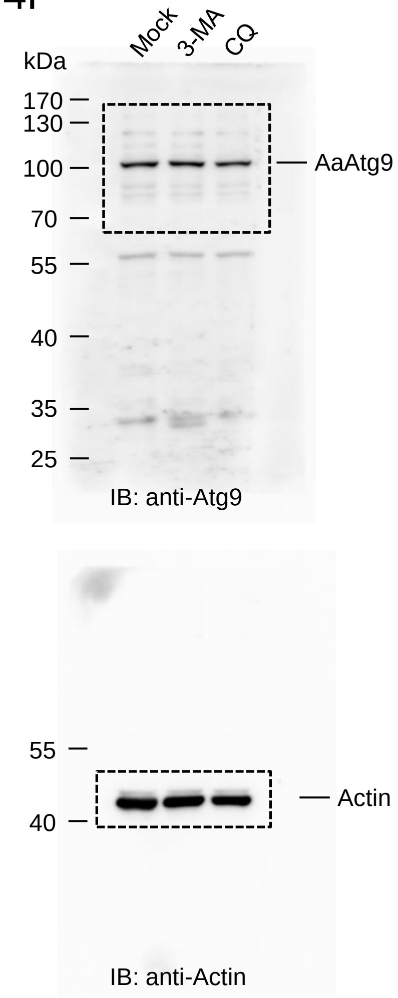

Fig 4G

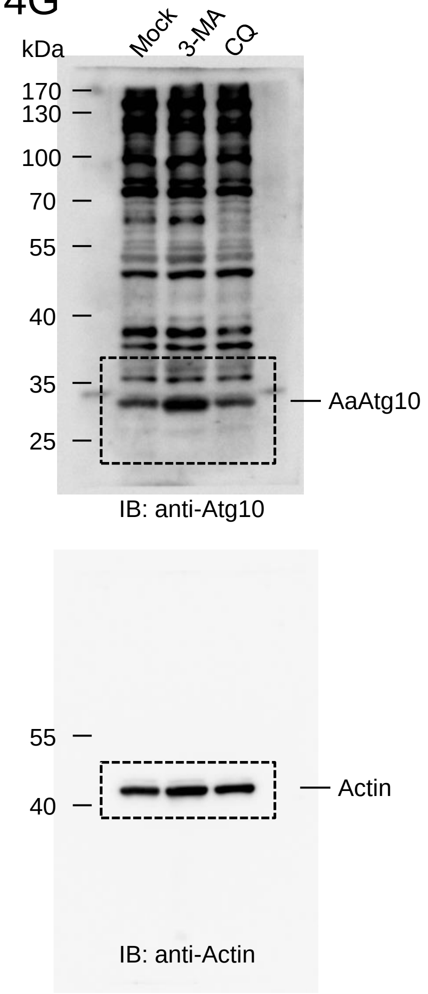

Fig 4H

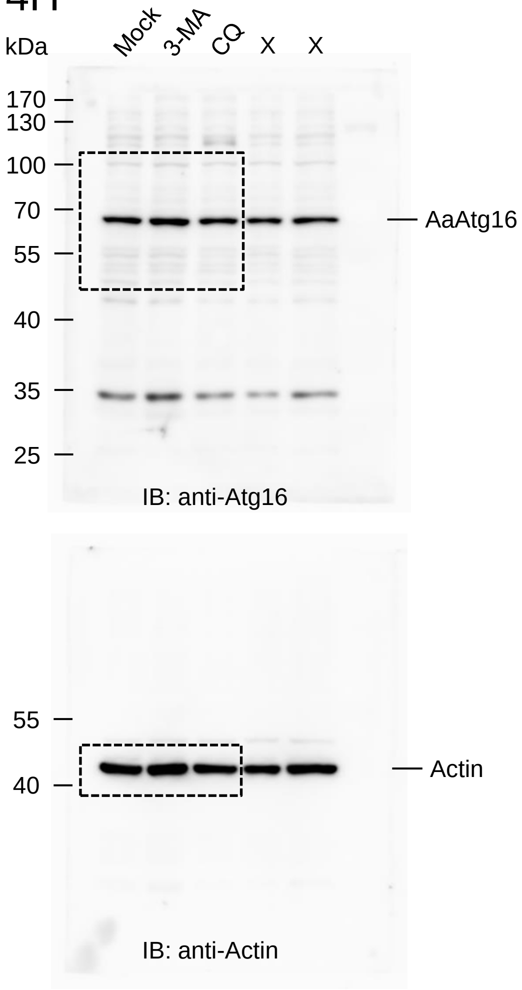

Fig 4I

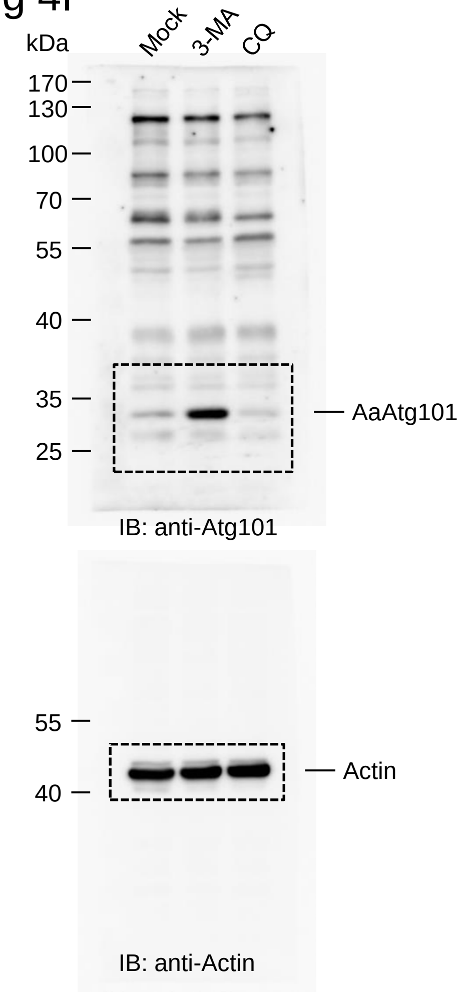

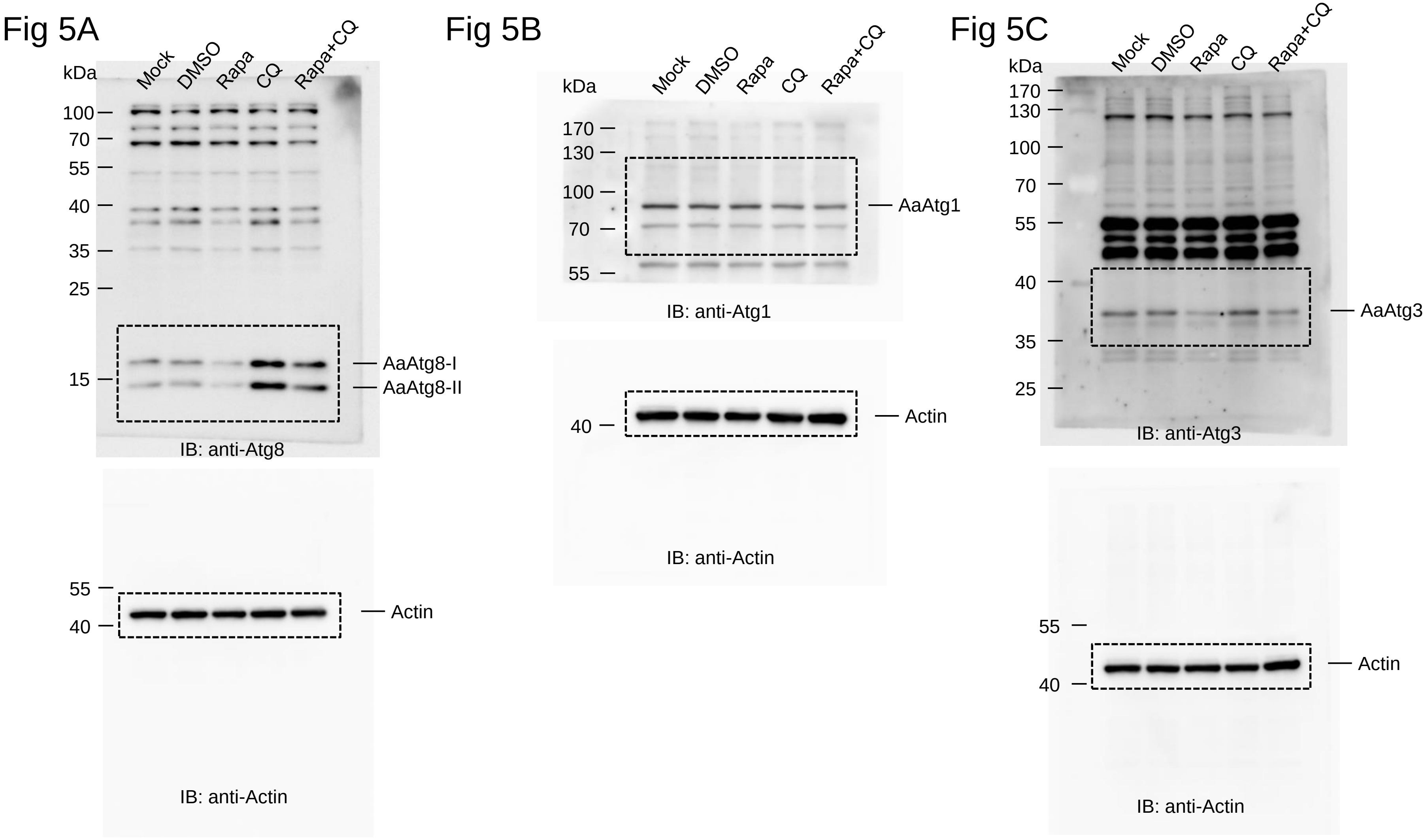

Fig 5A
Mock
DMSO
Rapa
CQ
Rapa+CQ
kDa
100
70
55
40
35
25
15
AaAtg8-I
AaAtg8-II
IB: anti-Atg8
55
40
Actin
IB: anti-Actin
Fig 5B
Mock
DMSO
Rapa
CQ
Rapa+CQ
kDa
170
130
100
70
55
AaAtg1
IB: anti-Atg1
40
Actin
IB: anti-Actin
Fig 5C
Mock
DMSO
Rapa
CQ
Rapa+CQ
kDa
170
130
100
70
55
40
35
25
AaAtg3
IB: anti-Atg3
55
40
Actin
IB: anti-Actin

Fig 5D

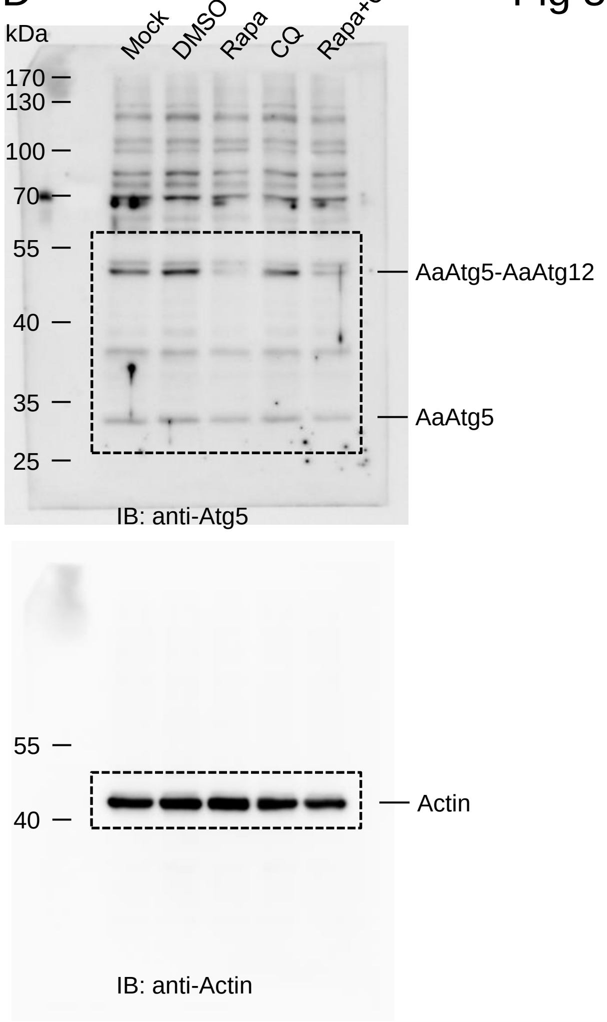

Fig 5E

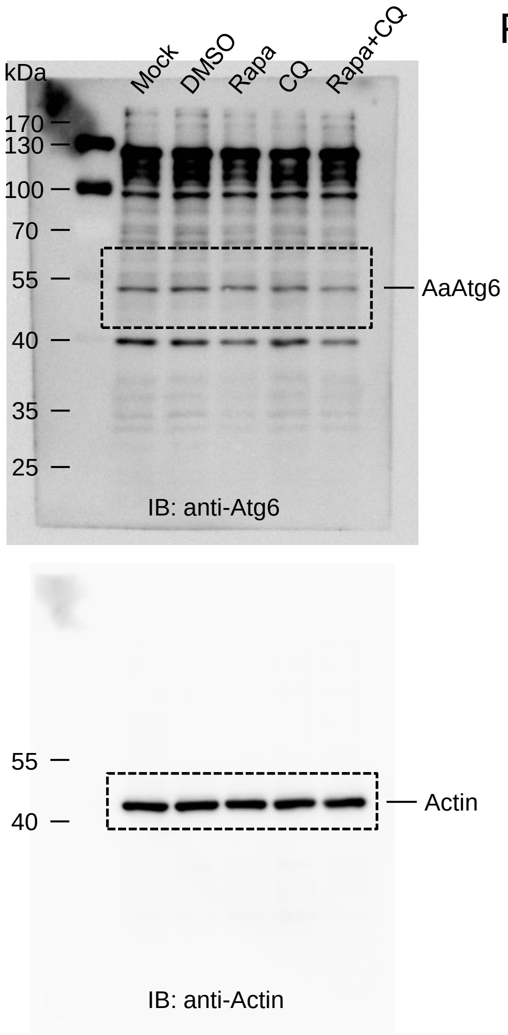

Fig 5F

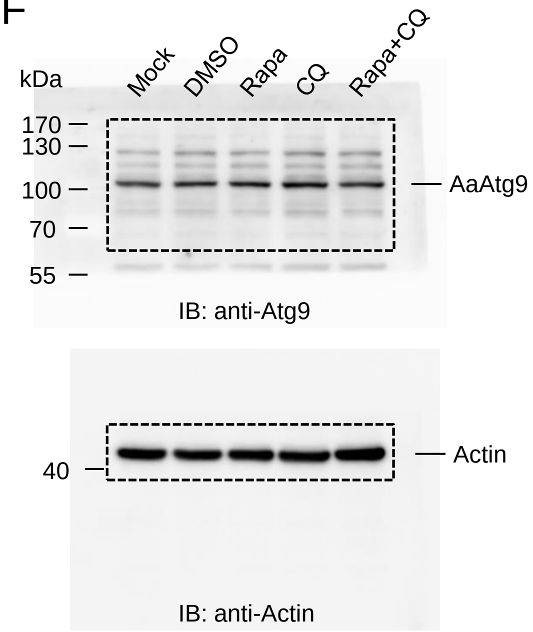

Fig 5G

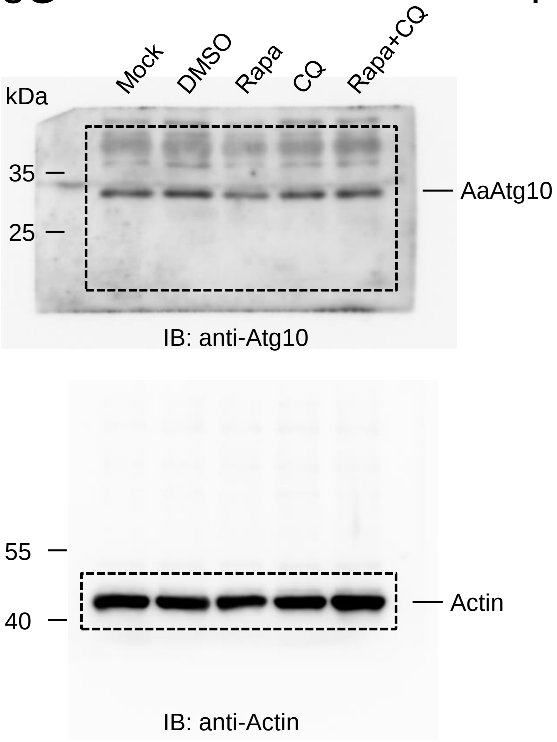

Fig 5H

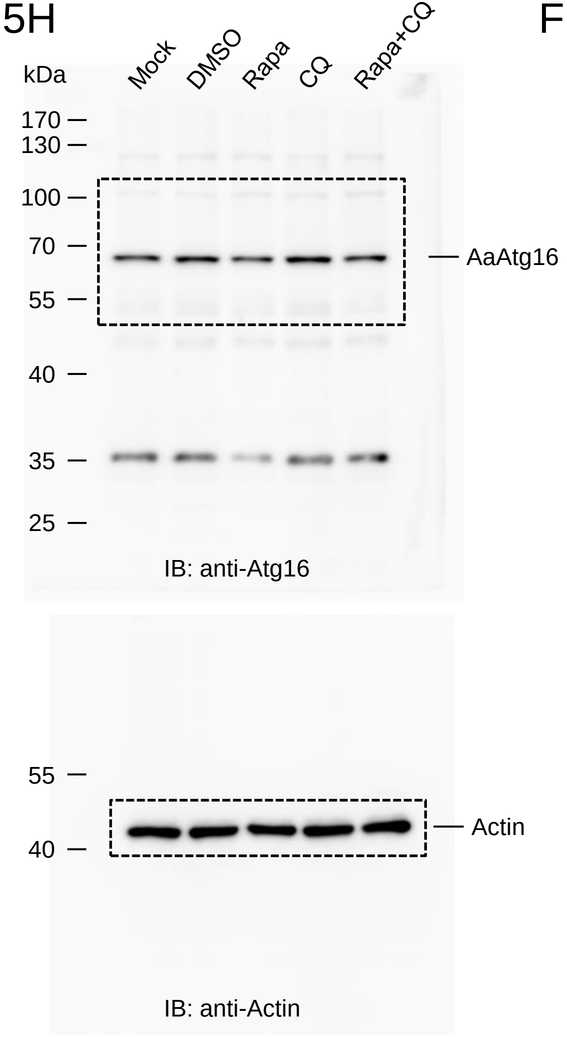

Fig 5I

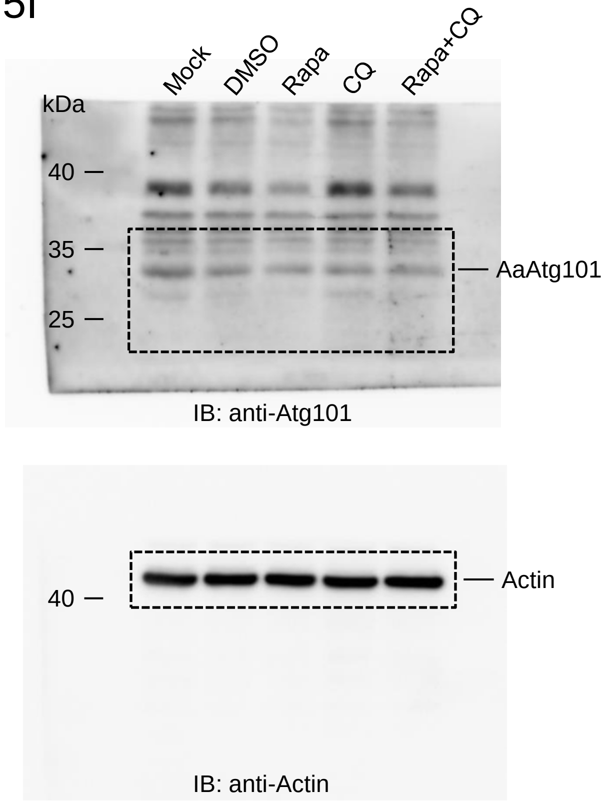

Fig 6B

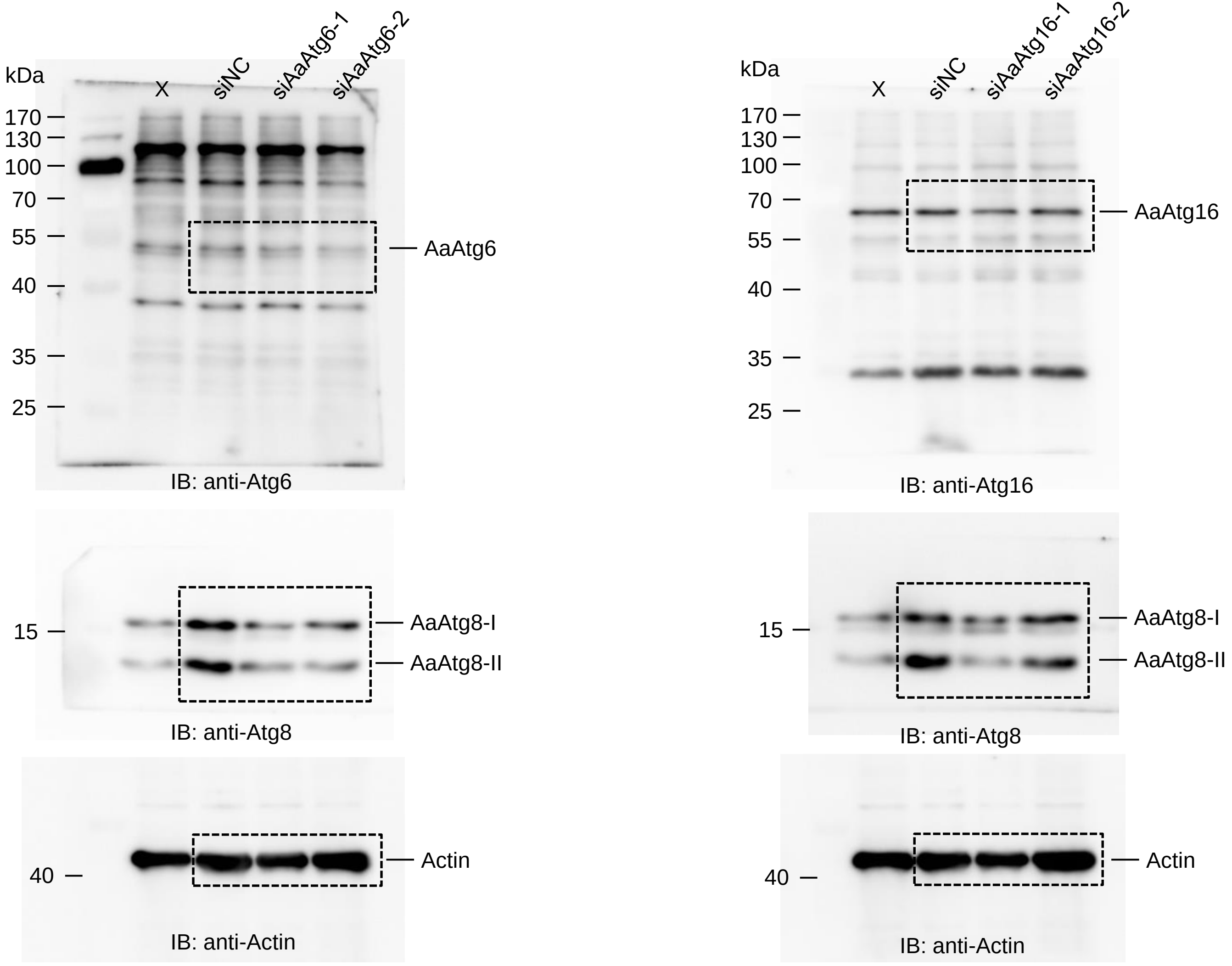
kDa
X
siNC
siAaAtg6-1
siAaAtg6-2
170
130
100
70
55
40
35
25
AaAtg6
IB: anti-Atg6
15
AaAtg8-I
AaAtg8-II
IB: anti-Atg8
40
Actin
IB: anti-Actin
kDa
X
siNC
siAaAtg16-1
siAaAtg16-2
170
130
100
70
55
40
35
25
AaAtg16
IB: anti-Atg16
15
AaAtg8-I
AaAtg8-II
IB: anti-Atg8
40
Actin
IB: anti-Actin

Fig 6C

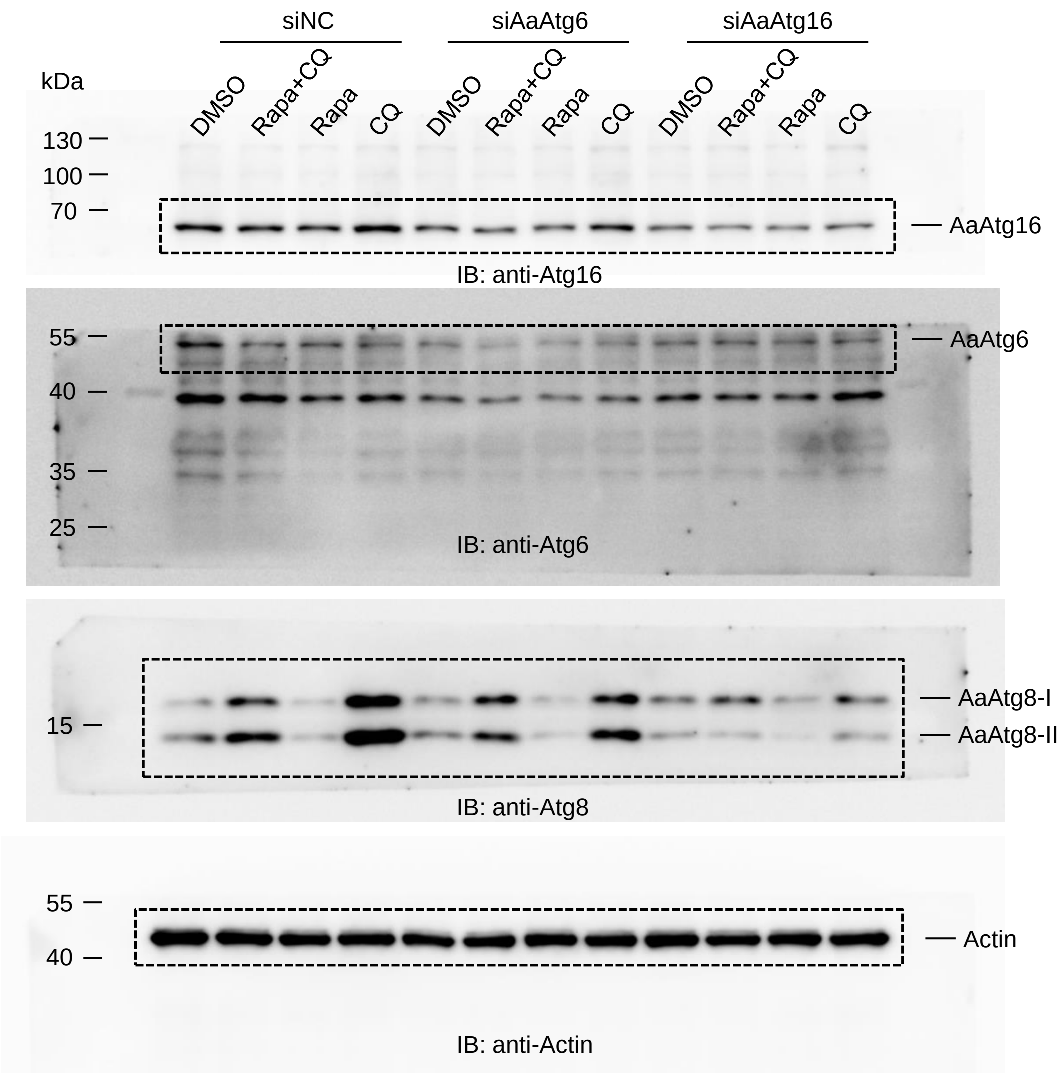

siNC
siAaAtg6
siAaAtg16
kDa
DMSO
Rapa+CQ
Rapa
CQ
130
100
70
AaAtg16
IB: anti-Atg16
55
40
35
25
AaAtg6
IB: anti-Atg6
15
AaAtg8-I
AaAtg8-II
IB: anti-Atg8
55
40
Actin
IB: anti-Actin

Fig 7A

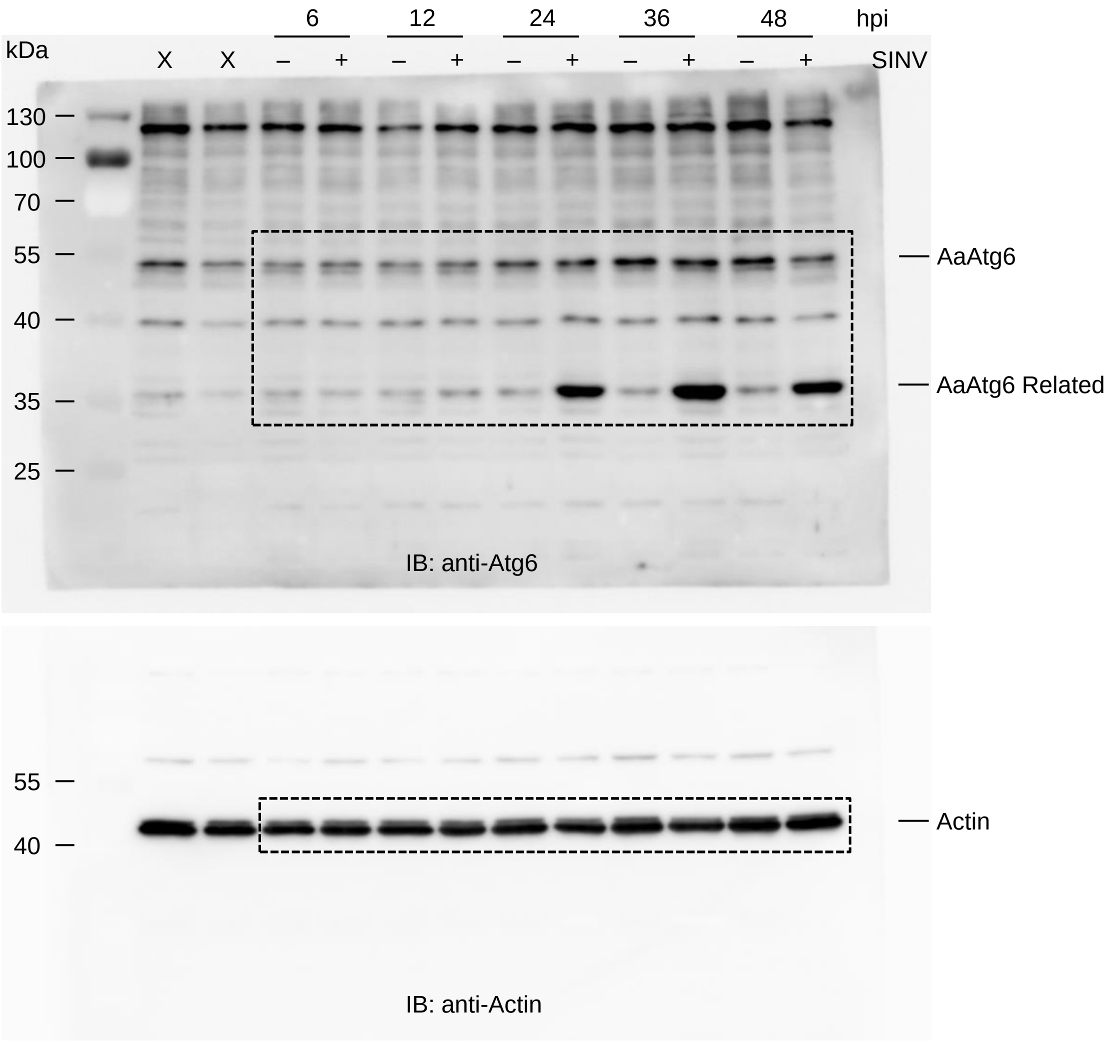

6
12
24
36
48
hpi
kDa
X X – + – + – + – + – +
SINV
130
100
70
55
40
35
25
AaAtg6
AaAtg6 Related
IB: anti-Atg6
55
40
Actin
IB: anti-Actin

Fig 7B

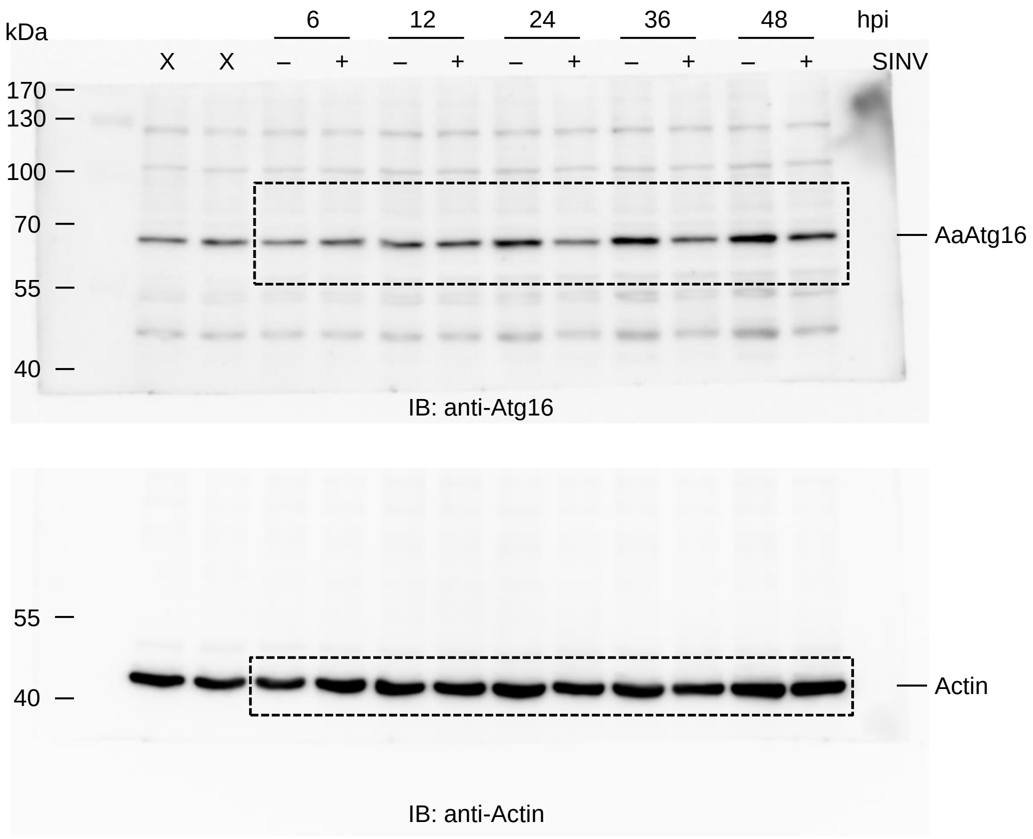

kDa
6
12
24
36
48
hpi
X
X
–
+
–
+
–
+
–
+
–
+
SINV
170
130
100
70
55
40
AaAtg16
IB: anti-Atg16
55
40
Actin
IB: anti-Actin
